# The microRNAs *miR-204* and *miR-211* maintain joint homeostasis and protect against osteoarthritis progression

**DOI:** 10.1038/s41467-019-10753-5

**Published:** 2019-06-28

**Authors:** Jian Huang, Lan Zhao, Yunshan Fan, Lifan Liao, Peter X. Ma, Guozhi Xiao, Di Chen

**Affiliations:** 1 Department of Orthopedic Surgery, Rush University Medical Center, Chicago, IL 60612 USA; 2 Department of Biologic and Materials Science, University of Michigan, Ann Arbor, MI 48109 USA

**Keywords:** miRNAs, Osteoarthritis

## Abstract

Osteoarthritis (OA) is a common, painful disease. Currently OA is incurable, and its etiology largely unknown, partly due to limited understanding of OA as a whole-joint disease. Here we report that two homologous microRNAs, *miR-204* and *miR-211*, maintain joint homeostasis to suppress OA pathogenesis. Specific knockout of *miR-204/-211* in mesenchymal progenitor cells (MPCs) results in Runx2 accumulation in multi-type joint cells, causing whole-joint degeneration. Specifically, *miR-204/-211* loss-of-function induces matrix-degrading proteases in articular chondrocytes and synoviocytes, stimulating articular cartilage destruction. Moreover, *miR-204*/*-211* ablation enhances NGF expression in a Runx2-dependent manner, and thus hyper-activates Akt signaling and MPC proliferation, underlying multiplex non-cartilaginous OA conditions including synovial hyperplasia, osteophyte outgrowth and subchondral sclerosis. Importantly, *miR-204*/-*211*-deficiency-induced OA is largely rescued by Runx2 insufficiency, confirming the *miR-204*/-*211-*Runx2 axis. Further, intraarticular administration of *miR-204*-expressing adeno-associated virus significantly decelerates OA progression. Collectively, *miR-204*/*-211* are essential in maintaining healthy homeostasis of mesenchymal joint cells to counteract OA pathogenesis.

## Abstract (web summary)

Osteoarthritis involves whole-joint tissue degeneration. Here, the authors show that miR-204 and miR-211 in mesenchymal joint cells regulate their proliferation, catabolic and osteogenic responses, and that disease progression is ameliorated by intra-articular miR-204 delivery in mice.

## Introduction

Osteoarthritis (OA) is a painful joint disease affecting more than 10% of the adult population^1,2^. Pathological changes of OA embody multiple manifestations such as articular cartilage (AC) destruction, synovial hyperplasia, osteophyte formation, and subchondral bone sclerosis. In addition, OA patients often show varied symptoms and respond differently to clinical interventions, suggesting that OA is a heterogeneous disease and its management demands therapeutics that offer greater precision^3,4^. Currently available studies ascribe the pathogenic causes of OA to dysregulation of anabolic and catabolic pathways that affect cartilage matrix maintenance and bone remodeling. However, clinical trials targeting related factors such as matrix metalloproteinases (MMPs), inflammatory cytokines, or growth factors, showed mixed results regarding the efficacy or safety of the therapeutics^5,6^. Thus, development of efficacious OA therapies calls for more in-depth understanding of OA and should benefit from identification of new molecular mechanisms that underlie the complex and multifaceted phenotypes of OA.

Multiple joint tissues undergo structural and functional failure during OA pathogenesis, including AC, meniscus, and synovium. These tissues are mesenchymal or contain significant portions of mesenchymal cells. Moreover, mesenchymal stem cells (MSCs) and mesenchymal progenitor cells (MPCs) are thought to exist in these joint tissues and play important roles in joint homeostasis and maintenance^7^. Importantly, the formation of osteophytes, a prominent radiographic feature of OA and a significant source of pain and joint dysfunction, requires MPCs derived from adjacent areas including periosteum and synovium to recapitulate endochondral ossification^8^. As miRNAs emerge as important regulators in skeletal pathophysiology^9,10^, their roles in MPC-derived skeletal tissues remain elusive. Through in vitro work we identified two homologous microRNAs, *miR-204* and *miR-211*, which negatively regulate Runx2 in MPCs^11^. However, the in vivo functions of *miR-204*/*-211* are largely unknown, especially regarding how they regulate MPCs and the pathophysiology of mesenchyme-derived skeletal tissues like synovial joints.

Here we identify a mechanism by which *miR-204*/*-211* regulate the proliferation and differentiation of MPCs in the entire joint and maintain healthy metabolism of joint tissues, thus helping the joint to counteract OA pathogenesis in a well-rounded manner. Our study takes advantage of in vivo analysis of genetically modified mice with *miR-204*/*-211* deficiency in MPCs and their descendent cells and we reveal that *miR-204*/*-211-*Runx2 axis is pivotal in maintaining joint tissue homeostasis. Dysregulation of this signaling pathway leads to multifactorial pathogenesis of OA, particularly with respect to noncartilaginous pathological anabolic changes such as synovial hyperplasia and osteophyte formation. In addition, our results demonstrate that intraarticular reintroduction of *miR-204* alleviates OA progression in a surgically induced OA model.

## Results

### Global deficiency of *miR-204/-211* induces OA

**Fig. 1:**
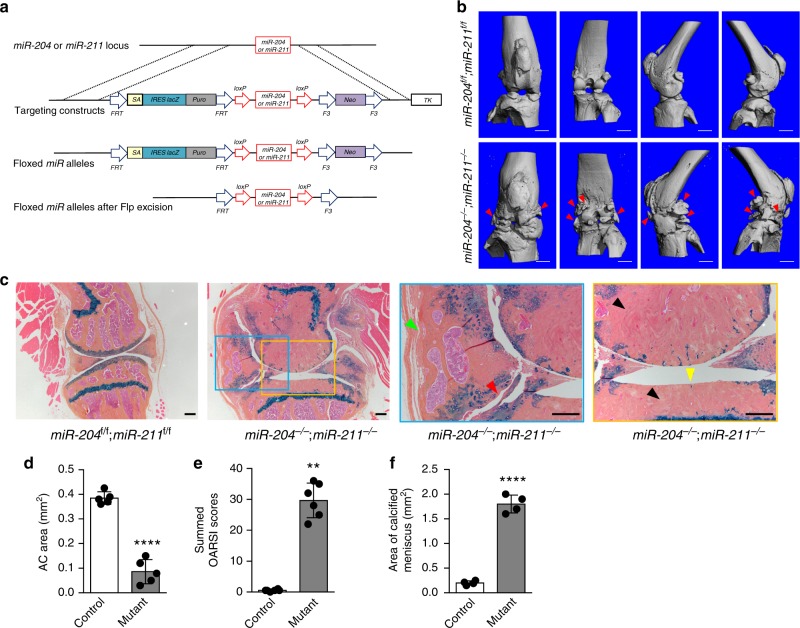
Germline deletions of *miR-204*/*-211* cause multifaceted OA phenotype. **a** Schematic of *miR-204* (or *miR-211*) conditional knockout (cKO) allele. Note that the contents, such as lines, boxes and arrows, are not proportional. **b** Representative μCT images of 46-week-old *miR-204*/*-211* floxed (control) and global double knockout mice (*miR-204*^−/−^; *miR-211*^−/−^). Red arrowheads, osteophytes. *n* = 5. Scale bar, 1 mm. **c** Representative histology images of knee joints of 46-week-old *miR-204*/*-211* floxed mice and global double KO (*miR-204*^−/−^; *miR-211*^−/−^) mice stained by Alcian blue/Orange G. Yellow arrowhead, loss of articular cartilage; red arrowhead, osteophytes; green arrowhead, synovial hyperplasia; black arrowheads, subchondral sclerosis. *n* = 5. Scale bar, 200 μm. **d**, **f** Histomorphometric quantification of medial AC area (**d**), and calcified meniscus area (**f**) of 46-week-old *miR-204*/*-211* floxed mice (control) and *miR-204*^−/−^; *miR-211*^−/−^ mice (mutant). ****P < 0.0001, *n* = 4 or 5. Unpaired Student’s *t* test. **e** Osteoarthritis research society international (OARSI) scoring system was used to evaluate knee joint articular cartilage (AC) destruction in 46-week-old control and mutant mice. **P < 0.01, Mann−Whitney test. *n* = 6

Our in vitro data showed that two homologous *miRNAs*, *miR-204* and *miR-211*, suppress Runx2 expression in MPCs^11^. However, little is known about their in vivo functions. Thus, we have generated mouse models with conditional knockout (cKO) alleles for *miR-204* and *miR-211*. A diagram of the targeting vector for *miR-204* or *miR-211* is shown in Fig. 1a. To remove the *lacZ* and *Neo* cassettes, we crossed the targeted mice with flippase-expressing transgenic mice, and thus generated floxed alleles for the *miRNAs* (Fig. 1a, Supplementary Fig. 1a). Since *miR-204 or miR-211* was reported to be expressed in a variety of tissues and play diverse pathophysiological roles^12–16^, we were interested to investigate if genetically modified animals with global deletions of both *miRNAs*, which were not yet available but are essential to elucidation of the *miR-204*/*-211* functions, showed any aberrant phenotypes. Thus, we crossed CMV-Cre transgenic mice^17^ with *miR-204*^*flox*/*flox*^ and *miR-211*^*flox*/*flox*^ mice to generate global knockout (KO) of both *miRNAs* (Supplementary Fig. 1b, c), namely the mice genotyped as *miR-204*^*−*/−^; *miR-211*^*−*/−^ mice. These *miR-204*/*-211*-deficient mice did not show very apparent abnormality postnatally and postpubertally. However, our preliminary analysis of older global *miR-204*/*-211* KO mice through the Mouse Metabolic Phenotyping Center at University of California, Davis has revealed a spectrum of pathological changes such as hypermature cataracts with rupture, uveal melanosis and melanosis, retinal dysplasia, follicular dysplasia, valvular endocardiosis, etc. (Supplementary Table 1).

We also looked at the knee joint phenotype of the global KO mice. At the age of 9 weeks, the *miR-204*^−/−^; *miR-211*^−/−^ mice did not differ from the wild type in joint histology, particularly in terms of the tibial growth plate length and AC thickness (Supplementary Fig. 1d, f, g). At the age of 15 weeks, the *miR-204*^−/−^; *miR-211*^−/−^ mice still did not show difference in growth plate length, but started to possess increased AC abrasion (Supplementary Fig. 1e, h). Strikingly, examination of the aged *miR-204*^−/−^; *miR-211*^−/−^ mice showed swollen knee joints, which was demonstrated by μCT analysis to contain ectopically grown bone spurs, resembling osteophyte outgrowth in human OA patients (Fig. 1b). The structural deterioration in the knee joints of the mutant mice involved multiple types of tissues inside the joint, as displayed by highly abrasive AC, subchondral bone thickening, severe synovial hyperplasia, excessive osteophyte formation, and meniscus calcification (Fig. 1c−f). As the pathologic condition of human OA disease and a well-established OA model^18^ (Supplementary Fig. 1i) embodies all of these histological changes, we concluded that the *miR-204*/*-211-*deficient mice had undergone OA onset and progression.

### Deletion of *miR-204/-211* in MPCs promotes OA pathogenesis

The tissues affected by OA pathogenesis in the *miR-204*/*-211* KO mice include AC, meniscus, subchondral bone, synovium and joint capsules, of which all are mesenchymal tissues. This suggested that the phenotype observed in the *miR-204*/*-211* KO mice could be caused by the loss of *miR-204*/*-211* in the mesenchymal stem/progenitor cells, which give rise to these joint tissues. Thus, we decided to specifically delete *miR-204*/*-211* in MPCs by crossing the *miR-204*^*flox*/*flox*^ and *miR-211*^*flox*/*flox*^ mice with *Prx1-Cre* transgenic mice, in which the *Prx1* promoter was reported to drive Cre expression in the early limb bud mesenchyme and in the MPCs^19,20^.

Single KO mice for *miR-204* (*miR-204*^*flox*/*flox*^; *Prx1-Cre*) or *miR-211* (*miR-211*^*flox*/*flox*^; *Prx1-Cre*) did not show obvious joint phenotype (Supplementary Fig. 2a, b). Thus, we focused on breeding and analyzing double KO mice (*miR-204*^*flox*/*flox*^; *miR-211*^*flox*/*flox*^; *Prx1-Cre*, hereafter dKO). Interestingly, we had observed a significantly lower birth ratio of the dKO mice than that of the littermates without *Prx1-Cre*. This prompted us to perform a whole-genome sequencing, which revealed that the *Prx1-Cre* transgene is located on Chromosome 7 (Supplementary Fig. 2c) where *miR-211* also resides. Thus, the lower ratio of the dKO mice was largely contributed by the genetic linkage between *miR-211* and transgenic *Prx1-Cre*. Meanwhile, we have not observed significant gross abnormality of the dKO pups, which survived as well as their *Prx1-Cre*^*−*^ littermates, suggesting that the ablation of *miR-204*/*-211* has no apparent impacts on skeletal development and growth in the young mice.

**Fig. 2:**
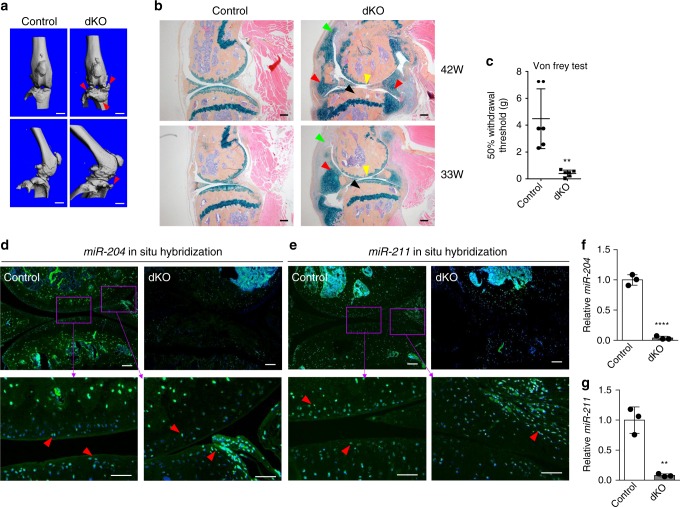
Deficiency of *miR-204*/-*211* in mesenchymal progenitor cells recapitulates OA pathogenesis. **a** Representative μCT images of 42-week-old *miR-204*/*-211* floxed (control) and conditional double KO (dKO, with *Prx1-Cre*) mice. Red arrowheads, osteophytes. *n* = 5. Scale bar, 1 mm. **b** Representative histology images of knee joints in 42- (top) or 33-week-old (bottom) control and dKO mice stained by Alcian blue/Orange G. Yellow arrowheads, loss of articular cartilage; red arrowheads, osteochondrophytes; green arrowheads, synovial hyperplasia; black arrowheads, subchondral sclerosis. *n* = 5. Scale bar, 200 μm. **c** Results of von Frey test on 33-week-old control and dKO mice. *n* = 6. **P < 0.01, unpaired Student’s *t* test. **d**, **e** In situ hybridization using highly specific LNA-enhanced *miR-204* (**d**) or *miR-211* (**e**) probe (green) on knee joint sections of 7-month-old mice. The bottom panels are the enlargements of the upper images as indicated. DAPI staining marks nuclei (blue). Red arrowheads, *miR-204-* or *miR*-*211*-positive cells. Scale bar, 100 μm. **f**, **g** qRT-PCR analysis of *miR-204* and *miR-211* expression in CD45^−^ BMSCs from control or dKO mice. **P *<* 0.01, ****P < 0.0001, unpaired Student’s *t* test. *n* = 3. Data are shown as the mean ± s.d.

We examined the dKO mice to identify if the OA phenotype caused by a global deficiency of *miR-204*/*-211* could be recapitulated by the loss of the *miRNAs* in MPCs. The μCT scanning of the knee joints of 42-week-old mice revealed striking osteophyte growth (Fig. 2a). Further histology analysis of the dKO mice confirmed strong OA-like phenotypes, including synovial hyperplasia, cartilage degradation, subchondral sclerosis, osteophyte formation, and narrowed knee joint space (Fig. 2b). Particularly, the OA phenotypes developed gradually with age, as exemplified by 42-week-old mice showing more severe synovial hyperplasia and cartilage degradation than 33-week-old mice (Fig. 2b). It is notable that osteophytes were accompanied by more ectopic cartilage formation (blue staining) in 33-week-old mice than in 42-week-old mice, suggesting that osteophyte growth was preluded by excess cartilage formation (Fig. 2b). In other words, osteophyte growth in the dKO knee joints resulted from de novo endochondral ossification that requires cartilaginous templates derived from MPCs. Osteoarthritis Research Society International (OARSI) scoring^21^ also demonstrated more severe cartilage destruction in the dKO mice and a progressive OA development (Supplementary Fig. 2d). As pain is the most common individual burden for OA patients and the most prominent symptom of OA, we also performed behavioral pain tests and found that the dKO mice had increased pain sensitivity compared with *Prx1-Cre*-negative littermates (Fig. 2c). Thus, our results suggested that the deficiency of *miR-204*/*-211* in MPCs and mesenchymal joint tissues contributed to the genesis of OA pain, possibly through the structural deterioration of the joint.

Next, we performed in situ hybridization (ISH) assays with highly specific locked nucleic acid (LNA)-enhanced miRNA probes to determine the expression of *miR-204* and *miR-211* in the control and dKO mice. Our results demonstrated that both *miR-204* and *miR-211* are expressed in knee joint tissues, such as AC, meniscus and synovium, which all have mesenchymal origin (Fig. 2d, e, Supplementary Fig. 2e, f). As well, immunohistochemistry (IHC) assays showed that the *Prx1* promoter drives Cre expression in AC, meniscus and synovium, suggesting a comprehensive deletion of *miR-204*/*-211* in joint tissues by *Prx1-Cre* (Supplementary Fig. 2g). Significantly diminished ISH signals of *miR-204* (or *miR-211*) were displayed in AC, synovium and meniscus of dKO mice (Fig. 2d, e), suggesting that *miR-204* and *miR-211* had been effectively and specifically deleted in multiple types of mesenchymal tissues in the joint. Moreover, quantitative RT-PCR (qRT-PCR) assays demonstrated that both *miR-204* and *miR-211* significantly decreased in RNAs extracted from AC or synovium (Supplementary Fig. 2h, i), while the gene expression of the *miRNA* host genes (*Trpm3* and *Trpm1*) had no significant change (Supplementary Fig. 2j, k). To further confirm the ablation of *miR-204*/*-211* in other mesenchymal tissues particularly bone marrow MPCs, we also isolated and cultured CD45^−^ bone marrow stromal cells (BMSCs), of which the major fraction was shown to be MPCs^22^ (Supplementary Fig. 3). Subsequent qRT-PCR experiments showed a 90% reduction of *miR-204*/*-211* in CD45^−^ BMSCs isolated from the dKO mice (Fig. 2f, g). Together, our results confirmed that *miR-204*/*-211* are effectively deleted in mesenchymal tissues, including AC, meniscus and synovium of the knee joints.

### Deficiency of *miR-204/-211* elevates Runx2 and OA markers

The CD45^−^ BMSCs deficient of *miR-204*/*-211* may be used for the confirmation of the interacting machinery between *miR-204*/*-211* and *Runx2* mRNA at the molecular level. Thus, we applied the cross-linking and immunoprecipitation (CLIP) technique^23^ to purify the complex comprising Argonaute (AGO) proteins, Ago-bound *miRNAs* and *miRNA*-targeting mRNAs^24^. Our results demonstrated that *miR-204*/*-211* bind to *Runx2* mRNA at two binding sites (Supplementary Fig. 4a). Furthermore, ablation of *miR-204*/*-211* in CD45^−^ BMSCs significantly decreased the ligated chimera between *miR-204*/*-211* and *Runx2* mRNA (Supplementary Fig. 4b), suggesting that *miR-204*/*-211* deletion impaired the formation of the AGO-*miR-204*/*-211*-*Runx2* mRNA tertiary complex and therefore relieved Runx2 suppression by *miR-204*/*-211*.

**Fig. 3:**
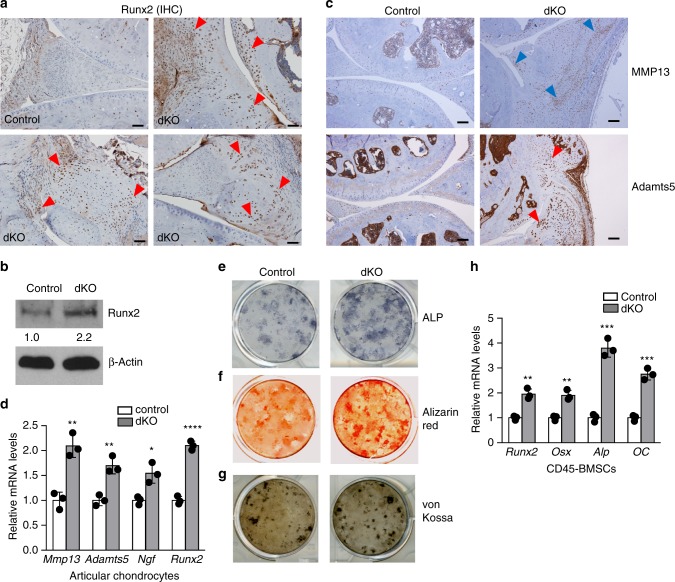
Deficiency of *miR-204*/*-211* upregulates Runx2, cartilage-catabolic enzymes and osteogenic markers. **a** IHC results show enhanced Runx2 expression in the synovium, articular cartilage (AC), and meniscus of knee joints of dKO mice. Red arrowheads, Runx2-positive cells. Scale bar, 50 μm. **b** Western blot analysis shows Runx2 accumulation in CD45^−^ BMSCs isolated from dKO mice. The numbers below the blot indicate the relative protein levels of Runx2 (normalized to β-actin) in each group compared to the control. *n* = 3. **c** IHC results of MMP13 and Adamts5 in control and dKO mice. Arrowheads, IHC-positive cells. Scale bar, 100 μm. **d** qRT-PCR analysis of *Mmp13*, *Adamts5*, *nerve growth factor* (*NGF*), and *Runx2* expression in articular chondrocytes from control or dKO mice. Unpaired Student’s *t* test. *n* = 3. Data are shown as the mean ± s.d. **e**−**g** Alkaline phosphatase (ALP) staining (**e**), Alizarin red staining (**f**), and von Kossa staining (**g**) of BMSCs isolated from control or dKO mice. *n* = 3. **h** qRT-PCR analysis of *Runx2*, *Osterix* (*Osx*), *Alp* and *Osteocalcin* (*OC*) expression in CD45^−^ BMSCs from control or dKO mice. Unpaired Student’s *t* test. *n* = 3. Data are shown as the mean ± s.d. *P < 0.05, **P *<* 0.01, ***P < 0.001, ****P < 0.0001. Uncropped western blot scans are shown in Supplementary Data 1

In addition, our IHC results showed that protein levels of Runx2 were significantly elevated in AC, meniscus and synovium, especially in thickened synovium and osteophyte tissues (Fig. 3a, Supplementary Fig. 4c). Besides mesenchymal tissues in knee joints, CD45^−^ BMSCs of the dKO mice also showed higher expression of Runx2 proteins (Fig. 3b). These results collectively suggest that *miR-204*/*-211* deletion in MPCs caused significant accumulation of Runx2 in a variety of MPC-derived mesenchymal tissues. Runx2 has been reported to induce matrix-degrading enzymes such as MMP13, Adamts5 and Adamts4^25–28^, which are upregulated during OA development and therefore deemed as OA marker genes. Thus, we performed IHC to determine if the OA phenotype in the dKO mice is associated with upregulation of cartilage-degrading enzymes. Our results showed that protein levels of MMP13, Adamts5 (Fig. 3c, Supplementary Fig. 4d, e) and Adamts4 (Supplementary Fig. 4f, g) were significantly elevated in joint tissues, such as AC, meniscus and synovium, of the dKO mice. In addition, we extracted total RNA from AC of knee joint and found that mRNA levels of *Mmp13* and *Adamts5* increased in the dKO mice, suggesting that *Mmp13* and *Adamts5* were upregulated at transcriptional level (Fig. 3d), possibly due to elevated Runx2 levels. Together, our results suggest that the ablation of *miR-204* and *miR-211* in mesenchymal tissues causes Runx2 accumulation and subsequent induction of proteases, including MMP13, Adamts5 and Adamts4, which mediate catabolic activities in AC degradation and contribute to OA pathogenesis.

OA is a complex degenerative joint disorder involving not only cartilage catabolism but also anabolic effects including osteophyte formation and subchondral bone sclerosis, two remarkable exhibitions of aberrantly enhanced osteogenesis. As Runx2 is a master regulator in bone formation, we asked if skeletal stem/progenitor cells from the dKO mice, which showed higher Runx2 levels than the cells from the control mice (Fig. 3b), possessed augmented osteogenic activity. We isolated and cultured BMSCs for alkaline phosphatase (ALP), Alizarin red and von Kossa staining, and found that osteoblast differentiation in the cells with *miR-204*/*-211* deletion accelerated (Fig. 3e−g). We also purified CD45^−^ BMSCs from the dKO mice and found that the mRNA levels of osteoblast markers such as *Osterix*, *Alp* and *osteocalcin* increased markedly in the dKO cells (Fig. 3h), suggesting that loss-of-function of *miR-204*/*-211* in skeletal progenitor cells promotes osteoblast differentiation. Combined with the IHC data that demonstrated Runx2 accumulation in joint tissues, our results suggest that increased subchondral bone formation and osteophyte outgrowth could result from enhanced Runx2 levels and elevated osteogenic activity, which are mediated by *miR-204*/*-211* deletion in mesenchymal tissues.

### Induced MPC proliferation/synovial hyperplasia in dKO joints

**Fig. 4:**
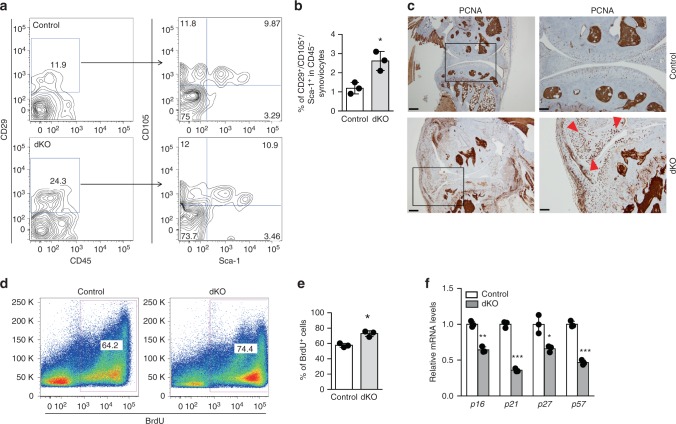
Proliferation of mesenchymal stem/progenitor cells is enhanced in *miR-204*/-*211* dKO mice. **a**, **b** Representative flow cytometry profile (**a**) and quantification (**b**) of CD29^+^/CD105^+^/Sca-1^+^ cells in CD45^−^ synoviocytes freshly isolated from control and dKO mice. *n* = 3. **c** IHC results of proliferating cell nuclear antigen (PCNA) in control and dKO mice. Red arrowheads, PCNA-positive cells. Scale bars (left panels), 250 μm. Scale bars (right panels), 100 μm. **d**, **e** Representative flow cytometry profile (**d**) and quantification (**e**) of BrdU-labeled CD45^−^ cells, which were in vivo labeled and freshly isolated. *n* = 3. **f** Quantitative RT-PCR analysis of *p16*, *p21*, *p27* and *p57* expression in CD45^−^ BMSCs from control or dKO mice. *n* = 3. Unpaired Student’s *t* test. Data are shown as the mean ± s.d. *P *<* 0.05, **P *<* 0.01, ***P *<* 0.001

We also observed synovial hyperplasia in the dKO mice, which represents an important pathological manifestation of OA. As fibroblast-like synovial cells are of mesenchymal origin and display MSC properties such as self-renewal and multipotentiality^29^, we investigated if the deficiency of *miR-204*/*-211* in MPCs caused aberrant proliferation of mesenchymal cells in synovium, which could underlie hypertrophy of nonhematopoietic tissues in synovium. Firstly, we isolated synovial cells from the dKO mice for flow cytometry to assess the frequency of MPCs in synovial cells. Four cell surface markers were used: CD45 is a hematopoietic cell marker while CD29, CD105 and Sca-1 are markers for MSCs^22^. The frequencies of CD29^+^CD105^+^Sca-1^+^ MSCs in CD45^−^ nonhematopoietic synoviocytes were significantly higher in the dKO mice than those in their Cre^−^ littermates (Fig. 4a, b), suggesting that *miR*-*204*/*-211*-deficient mesenchymal cells in synovium underwent increased proliferation. To assess cell proliferation in the joint tissues, we performed IHC to examine the level of proliferating cell nuclear antigen (PCNA) or Ki-67, and found that positive staining for PCNA and Ki-67 was significantly elevated in the dKO joint tissues, particularly in hypertrophied synovium, indicating excessive proliferation of synovial cells (Fig. 4c, Supplementary Fig. 5a, b). Also, we injected BrdU into the dKO mice and performed flow cytometry to evaluate the percentage of BrdU-positive mesenchymal cells in bone marrow. Our results showed that the frequencies of BrdU^+^CD45^−^ cells, which represent proliferating nonhematopoietic cells, were significantly higher in the dKO mice than in the control mice (Fig. 4d, e), suggesting that mesenchymal cells with the *miR-204*/*-211* deficiency retains an enhanced proliferative ability. As well, we observed that the frequencies of CD45^−^/CD29^+^/CD105^+^/Sca-1^+^ cells in bone marrow cells were also higher in the dKO mice (Supplementary Fig. 5c, d). Together, our results suggest that *miR-204*/-*211* ablation results in a greater proliferation rate of MPCs, which provides excessive mesenchymal cells and therefore contributes significantly to synovial hyperplasia in the dKO mice.

While our results demonstrated indispensable roles of *miR-204*/*-211* in modulating mesenchymal cell differentiation and catabolism to ameliorate cartilage degradation and ectopic bone formation, our data also revealed an unanticipated facet of *miR-204*/*-211* regulation that could be related to the proliferation of MPCs. To further investigate the mechanism altering the proliferation of the dKO cells, we analyzed mRNA expression of cyclin-dependent kinase (CDK) inhibitors, such as *p16*, *p21*, *p27* and *p57*, in CD45^−^ BMSCs derived from the dKO mice. Consistent with increased proliferation of mesenchymal cells as shown by BrdU labeling experiments, the mRNA levels of CDK inhibitors decreased substantially (Fig. 4f), suggesting that *miR-204*/*-211* loss-of-function has negative effects on CDK inhibitors to promote cell cycle progression in MPCs.

### Elevated Akt signaling and NGF expression in dKO joints

**Fig. 5:**
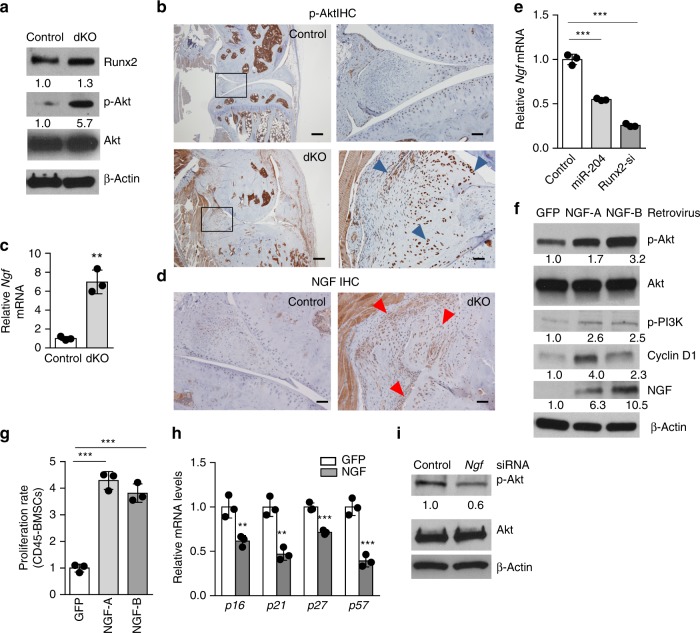
The *miR-204*/*-211*-Runx2 axis in regulation of NGF-Akt signaling and mesenchymal progenitor cell proliferation. **a** Western blot analysis shows activated Akt signaling in dKO CD45^-^ BMSCs. The numbers below the blot indicate the relative levels of the denoted protein (except p-Akt normalized to total Akt, all proteins were normalized to β-actin) in each group compared to the control. *n* = 3. **b** IHC results of p-Akt in control and dKO mice. Blue arrowheads mark p-Akt-positive cells. Scale bar, 250 μm (left) or 50 μm (right). **c** qRT-PCR analysis of *Ngf* mRNA expression in CD45^−^ BMSCs from control or dKO mice. **P *<* 0.01, unpaired Student’s *t* test. *n* = 3. **d** IHC results of NGF in control and dKO mice. Red arrowheads, NGF-positive cells. Scale bar, 50 μm. **e** qRT-PCR analysis of *Ngf* mRNAs shows that *miR-204* or *Runx2* siRNA decreases *Ngf* expression in CD45^−^ BMSCs. ***P *<* 0.001, one-way ANOVA followed by the Tukey−Kramer test. *n* = 3. **f** Retroviral overexpression of two NGF isoforms in CD45^−^ BMSCs from WT mice activates Akt signaling as shown by western blot. *n* = 3. **g** NGF overexpression increases proliferation of CD45^−^ BMSCs determined by cell number counts. ***P *<* 0.001, one-way ANOVA. *n* = 3. **h** qRT-PCR analysis of *CDK inhibitors* in mouse CD45^−^ BMSCs with GFP or NGF overexpression. **P *<* 0.01, ***P < 0.001, unpaired Student’s *t* test. *n* = 3. **i** Western blot analysis shows that *Ngf* knockdown downregulates Akt signaling in mouse CD45^−^ BMSCs. *n* = 3. All data are shown as the mean ± s.d. Uncropped western blot scans are shown in Supplementary Data 1

Because Akt signaling is reported to be pivotal for cell cycle regulation through blocking transcription of CDK inhibitors^30^, we performed western blot assays to determine if activation of Akt signaling is associated with enhanced proliferation of CD45^−^ BMSCs from *miR*-*204*/*-211*-deficient mice. We found that *miR-204*/*-211* ablation in CD45^−^ BMSCs dramatically increased the Thr308 phosphorylation of Akt proteins, while leaving the amount of total Akt proteins unchanged (Fig. 5a). We also carried out IHC on joint sections and found higher phospho-Akt level in the dKO mice than their Cre^−^ littermates, especially in hypertrophied synovium and ectopic bone/cartilage-like structures (Fig. 5b, Supplementary Fig. 6a), suggesting that Akt hyperactivation in mesenchymal tissues contributes to synovial hyperplasia and osteophyte outgrowth observed in the dKO mice.

Activation of Akt signaling cascade can occur with stimulation of receptor tyrosine kinases (RTK) by their ligands^31^. We found that mRNAs coding for nerve growth factor (NGF) were substantially upregulated in CD45^−^ BMSCs derived from dKO mice (Fig. 5c), which have been demonstrated to be comprised of mainly MPCs (Supplementary Fig. 3). The mRNA expression of *Ngf* is abundant in CD45^−^ BMSCs (ΔCt ≈ 5 compared to β-actin), suggesting that NGF is an endogenous player in mesenchymal cells. We also performed IHC on the dKO knee joint sections and found that NGF protein levels increased significantly in periarticular tissues, especially in synovium and newly formed ectopic cartilage/bone (Fig. 5d, Supplementary Fig. 6b), suggesting that aberrant increase of NGF, a major mediator of OA pain, is also associated with synovial hyperplasia and osteophyte formation in the dKO mice. To further confirm that NGF expression is induced by *miR-204*/*-211* loss-of-function and Runx2 accumulation, we introduced synthetic *miR-204* mimics or *Runx2* siRNAs into CD45^−^ BMSCs and found that both *miR-204* and *Runx2* siRNAs suppressed *Ngf* mRNA expression in CD45^−^ BMSCs (Fig. 5e), which may be mediated by the decrease of Runx2 levels by *miR-204* or by *Runx2* siRNAs (Supplementary Fig. 6c). These results indicate that *miR-204* and *miR-211* modulates NGF expression, Akt activation and cell proliferation through its regulation of Runx2. Retroviral overexpression of two NGF isoforms, i.e. NGF-A and NGF-B, both activated Akt signaling, as the results of western blots showed elevated levels of p-PI3K, p-Akt and cyclin D1 in NGF-transduced CD45^−^ progenitor cells (Fig. 5f). As well, NGF overexpression increased CD45^−^ cell proliferation (Fig. 5g). Importantly, the overexpression of NGF in CD45^−^ BMSCs significantly inhibited the mRNA expression of CDK inhibitors, such as *p16*, *p21*, *p27* and *p57* (Fig. 5h). Accordingly, the knockdown of *Ngf* by its siRNA transfection significantly reduced p-Akt protein levels and cell proliferation rate in CD45^−^ BMSCs (Fig. 5i, Supplementary Fig. 6d). Collectively, these results suggested that Runx2 accumulation induced by *miR-204*/*-211* depletion upregulates *Ngf* expression to activate Akt signaling and then to promote MPC proliferation, which may constitute a major source of excessive mesenchymal cells contributing to deleterious anabolic events in OA, including synovial hyperplasia, osteophyte outgrowth and subchondral sclerosis.

### Administration of *miR-204* alleviates surgery-induced OA

The dKO mice displayed severe, comprehensive OA phenotype, including AC degradation, subchondral sclerosis, osteophyte formation and synovial hyperplasia, suggesting that endogenous *miR-204*/*-211* play a key role in maintaining joint tissue homeostasis and preventing OA development. Thus, we ask if OA could be treated by administrating *miR-204*, as *miR-204* and *miR-211* share redundant functions and overexpression of one miRNA rescues the double knockout of two homologous miRNAs^32^. We performed partial meniscectomy to induce the onset of OA^33^, and then used intraarticular injection of adeno-associated virus (AAV) serotype 5^34^ to express *miR-204* in OA-inflicted knee joints. Three months after injection, we could still detect AAV expression in a variety of joint tissues including AC, meniscus and synovium (Supplementary Fig. 7), suggesting that AAV5 administered by intraarticular injection can drive potent and long-lasting gene expression in knee joints.

**Fig. 6:**
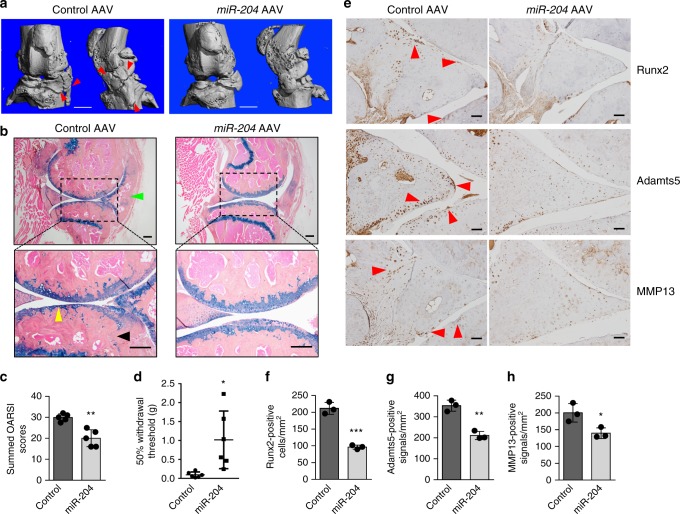
Intraarticular injections of AAV5-miR-204 alleviates surgically induced OA phenotype. **a** Representative μCT images of osteoarthritic joints in 9-month-old C57BL/6 mice receiving AAV5-GFP or AAV5-miR-204 injection at the age of 3 months. Red arrowheads, osteophytes. *n* = 5. Scale bar, 1 mm. **b** Representative histology images of osteoarthritic joints in 9-month-old mice receiving AAV injection at the age of 3 months. Yellow arrowheads, articular cartilage degradation; green arrowhead, synovial hyperplasia; black arrowhead, subchondral sclerosis. *n* = 5. Scale bars, 200 μm. **c** Analysis using OARSI scoring system was performed to evaluate knee joint AC destruction in mice receiving AAV5-GFP (control) or AAV5-miR-204 injection. *n* = 5. Mann−Whitney test. **d** Intraarticular injection of AAV5-miR-204 reduces pain sensitivity induced by OA. The von Frey test was performed in 9-month-old mice receiving AAV injection at the age of 3 months. *n* = 6. **e** IHC results of Runx2, Adamts5, and MMP13 expression in osteoarthritic joints receiving AAV injection. Red arrowheads, IHC-positive cells. Scale bar, 50 μm. **f**−**h** Quantification of positive IHC signals per mm^2^ of knee joints for detection of Runx2 (**f**), Adamtas5 (**g**), and MMP13 (**h**). *n* = 3. Unpaired Student’s *t* test. *P < 0.05, **P < 0.01, ***P < 0.001

Next, we performed phenotypic analysis to investigate how *miR-204* administration affects OA pathogenesis in the surgical OA model. Notably, μCT analysis showed that injection of AAV5-miR-204 diminished osteophyte growth significantly, compared with that of AAV5-control (Fig. 6a). Further, histological examination confirmed a substantial decrease of osteophyte formation and revealed marked mitigation regarding synovial hyperplasia and cartilage degradation in *miR-204*-injected joints (Fig. 6b, c). In pain-related behavioral tests, mice receiving *miR-204* injection showed higher pain thresholds than those receiving control AAV5 injection (Fig. 6d), suggesting that *miR-204* reintroduction in OA knee joints not only ameliorated histological features, including AC degradation and osteophyte formation, but also reduced pain, a prominent symptom affecting OA patients. We also used IHC to examine the protein levels of Runx2 and several OA markers, and found that *miR-204* administration alleviated the upregulation of Runx2, Adamts5 and MMP13, which was induced by the surgery (Fig. 6e−h). Together, our exploration of *miR-204* administration in vivo suggest that *miR-204* could act as an effective therapeutic agent to treat OA in a surgically induced OA mouse model, through inhibition of Runx2 expression in multiple joint tissues.

### The Runx2-*miR-204/-211* axis controls OA pathogenesis

**Fig. 7:**
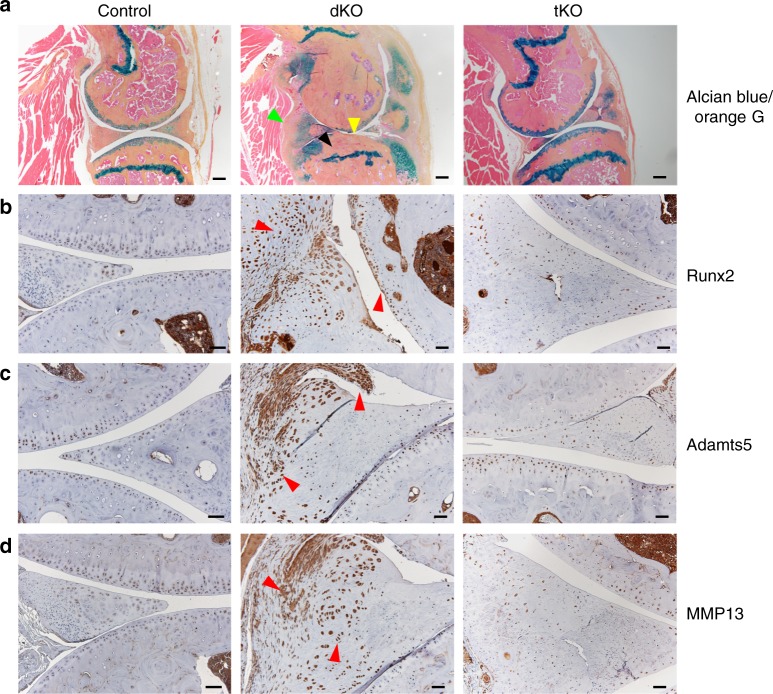
Runx2 insufficiency mitigates OA phenotype in dKO mice. **a** Representative histology images of 9-month-old tKO (*miR-204*^*flox*/*flox*^; *miR-211*^*flox*/*flox*^; *Runx2*^*+/flox*^; *Prx1-Cre*) mice compared with dKO (*miR-204*^*flox*/*flox*^; *miR-211*^*flox*/*flox*^; *Prx1-Cre*) and control (*miR-204*^*flox*/*flox*^; *miR-211*^*flox*/*flox*^) mice. Yellow arrowheads, articular cartilage degradation; green arrowhead, synovial hyperplasia; black arrowhead, subchondral sclerosis. *n* = 5. Scale bars, 200 μm. **b**–**d** IHC results of Runx2 (**b**), Adamts5 (**c**), and MMP13 (**d**) expression in the control, dKO and tKO mice. Red arrowheads, Runx2-, Adamts5-, or MMP13-positive cells. Scale bar, 50 μm

Accumulating evidence generated in this study points to *miR-204*/-*211*-regulated Runx2 that acts as a central player in OA onset and progression. To alleviate Runx2 accumulation caused by the ablation of *miR-204*/-*211* in skeletal cells, we introduced *Runx2* haploinsufficiency into the dKO mice by generating the *miR-204*^*flox*/*flox*^; *miR-211*^*flox*/*flox*^; *Runx2*^*+/flox*^; *Prx1-Cre* mice, hereafter tKO. Comparison of tKO and dKO mice revealed that the OA phenotype observed in the dKO mice has been largely ameliorated in the tKO mice. Specifically, the tKO mice possessed less degradation of AC in their knee joints than the dKO littermates, as demonstrated by histological analyses. Moreover, we have observed an effective diminish of synovial hyperplasia and osteophyte outgrowth in the tKO than in the dKO mice (Fig. 7a, Supplementary Fig. 8). These results suggest that Runx2 insufficiency can significantly mitigate the severe OA phenotype caused by *miR-204*/-*211* ablation in the skeletal cells.

To investigate if reduction of Runx2 protein levels was a causal factor in OA phenotype alleviation, we performed IHC assays to examine Runx2 protein levels in the tKO mice. Our results showed that Runx2 insufficiency markedly attenuated the Runx2 accumulating effects resulting from *miR-204*/-*211* deficiency, in AC, meniscus and synovium (Fig. 7b). As Runx2 level returned to normal as similar as that in the control mice, the tKO mice also showed a remarkable extenuation in cartilage-catabolic factors including Adamts5 and MMP13 compared to the dKO littermates (Fig. 7c, d). Thus, our results suggest that heterozygous deficiency of Runx2 rescued the OA phenotype resulting from *miR-204*/-*211* deletion and readjusted Runx2 proteins to a normal physiologic level, and therefore significantly mitigated the severe OA phenotype observed in the dKO mice. Further, we conclude that *miR-204*/-*211* control Runx2 expression and the Runx2-*miR-204*/-*211* axis governs OA pathogenesis.

## Discussion

Currently, many mechanistic OA studies focus on cartilaginous tissues^35–38^, since cartilage is a key component of synovial joints and cartilaginous tissues are exposed to mechanical stress that was thought to be a major risk factor for OA. Therefore, cartilage destruction is deemed to be a landmark event during OA onset and progression. In this study, we have generated a new mouse model in which *miR-204*/*-211* are deleted in multiple joint tissues and a comprehensive OA-like phenotype is observed in the entire joint but not restricted to cartilage only. For example, we found that the dKO mice develop severe synovial hyperplasia, which may have instrumental effects on other manifestations of OA, such as induced matrix-degrading enzymes on cartilage breakdown and elevated NGF on increased pain sensitivity and MPC proliferation. Osteophyte formation is also a prominent feature displayed in the dKO mice, and we found that osteophyte outgrowth is significantly contributed by excessive proliferation of MPCs, which implicates a mechanistic similarity between osteophyte formation in OA pathogenesis and endochondral ossification in skeletal development. Thus, our dKO mice could represent a comprehensive OA model, of which the analysis provides multifaceted insights into the mechanism of OA initiation and progression as well as the metabolic maintenance of the joint.

When we were generating the dKO mice, we had observed a much lower ratio of the dKO pups than that of the *miR-204*^*flox/flox*^;*miR-211*^*flox/flox*^ pups or the *miR-204*^*flox/flox*^;*miR-211*^*+/flox*^*;Prx1-Cre* pups. This made us wonder if significant prenatal lethality of the dKO mice occurred or the *Prx1-Cre* transgene is physically linked to *the miR-211* gene. Through whole-genome sequencing we have identified that the *Prx1-Cre* transgene is located on mouse Chromosome 7 (Supplemental Fig. 2c), the same chromosome on which the mouse *miR-211* gene also resides. Further we found that the ratio of the dKO pups was largely Mendelian if we crossed a dKO male to a *miR-204*^*flox/flox*^;*miR-211*^*flox/flox*^ female. These dKO mice did not show apparent abnormalities in young ages and their knee joint phenotypes were not significant at 3-month-old. However, the OA-like features of the dKO mice were aggravated with aging and the severity culminated at the ages of 10–12 months, which is milder than those genetic modified mouse models with disruption of major signaling pathways^39–41^, but bears greater resemblance to human OA. Thus, the use of the *Prx1* promoter and the *miR-204*/*-211* deletion at earlier stages in these dKO mice may not greatly affect their development and growth, suggesting that the OA phenotype observed at the later stages (>6 months) may be associated with accrual of *miR*-deficient effects in aging rather than in embryonic/developmental stages. On the other hand, an extensive deletion of *miR-204*/*-211* in MPCs as well as various types of mesenchymal cells inside the joints demonstrated multifaceted roles of *miR-204*/*-211* and generated comprehensive pathological changes not restricted to cartilage degradation. While the use of arguably more cartilage-specific promoters such as those of *Aggrecan* and *Col2a1* would provide cartilage-focused information for OA studies, *miRNA* ablation in MPCs offers a unique and resourceful perspective which help to advance our understanding of pathological changes of the entire joint.

OA can be regarded as a mesenchymal disease as it affects the entire synovial joint, which contains multiple connective tissues derived from MPCs, or in a broader context, MSCs. In addition, MSCs are reported to be available in most joint tissues, including synovium^42^, meniscus^43^ and cartilage^44^, which act as reservoirs of progenitor cells for tissue maintenance and repair as well as sources of paracrine signaling. Thus, the proper condition of MSCs is vital to the health of diarthrodial joints and OA pathogenesis involves dysregulation of MSC biology. In our study, *miR-204*/*-211* deficiency in MPCs causes detrimental alterations in both proliferation and differentiation, underlying multiple pathological anabolic properties of OA such as synovial hyperplasia, osteophyte outgrowth and subchondral sclerosis. Also, NGF overexpression in MPCs may constitute an important mechanistic cause for inappropriate nerve growth inside joints and resultant OA pain hypersensitivity. Due to the abilities of tissue regeneration and repair of MSCs which help to combat diseases such as OA and myocardial infarction as well as their immunomodulatory effects to alleviate immune disorders such as multiple sclerosis and Crohn’s disease^45^, the numbers of registered clinical trials of MSC-based therapies are rapidly increasing^46,47^. As our study demonstrated that *miR-204*/*-211* deficiency dysregulates the proliferation and differentiation of MPCs and thus causes OA phenotype, it also highlighted the importance of enhancing the understanding of MSC biology, which will help to develop safer MSC therapy and achieve better clinical outcomes.

In summary, our study of *miR-204*/*-211*-deficient mice suggests that the loss-of-function of *miR-204*/*-211* in MPCs leads to aberrant accumulation of Runx2, abnormally increases proliferation and osteogenic differentiation of MPCs, and causes comprehensive OA phenotype. Thus, our results suggest that *miR-204*/*-211* are essential for the joint to maintain healthy homeostasis and counteract OA pathogenesis.

## Methods

### Generation of *miR-204/-211* cKO mice

The animal protocol of this study has been approved by the Institutional Animal Care and Use Committee (IACUC) of the Rush University Medical Center and all experimental methods and procedures were carried out in accordance with the approved guidelines to comply with all relevant ethical regulations for animal testing and research. To generate *miR-204*/*-211* floxed mice, we engineered the targeting vectors for *miR-204* and *miR-211* using the recombineering-based method^48,49^. Briefly, an *IRES-lacZ* reporter cassette as wells as a *loxP* site were inserted 178 bp or 171 bp upstream *miR-204* or *miR-211* stem loop and a *loxP* site plus a Neomycin selection cassette were placed 260 bp or 365 bp downstream miRNA stem loops. Both *lacZ* and *Neo* cassettes are flanked by *Frt* or *F3* (a mutant of *Frt*) sites, allowing the subsequent removal of both cassettes by crossing the chimeric mice to flippase-expressing transgenic mice (The Jackson Laboratory, stock #: 003946)^50^ and leaving two loxP sites immediately flanking the miRNA stem-loops. Thus, the exogenous sequences brought by the targeting of miRNAs are minimal (146 nt at 5′ and 110 nt at 3′ for both floxed alleles) and did not affect the expression or intron splicing of host genes, *Trpm3* or *Trpm1*, of which *miR-204* or *miR-211* is located in the introns (data not shown). Floxed mice (*miR-204*^*flox*/*flox*^ or *miR-211*^*flox*/*flox*^) were backcrossed to C57BL/6 for over ten generations. To generate dKO mice (*miR-204*^*flox*/*flox*^; *miR-211*^*flox*/*flox*^; *Prx1-Cre*), male *Prx1-Cre* transgenic mice^19^ (The Jackson Laboratory, Bar Harbor, ME, USA) with the genotype of *miR-204*^*flox*/*flox*^; *miR-211*^*+*/*flox*^, namely *miR-204*^*flox*/*flox*^; *miR-211*^*+*/*flox*^*; Prx1-Cre*, were used to cross to female double floxed mice. To generate germline deletions of the *miRNAs*, *CMV-Cre* mice^17^ (The Jackson Laboratory, Bar Harbor, ME, USA) were used.

### AAV intraarticular injection and surgically induced OA model

We chose intraarticular injection of recombinant AAV for in vivo overexpression of *miR-204*. Both AAV5-EF1a-mmu-mir-204-GFP and AAV5-EF1a-ctrl-GFP were purchased from Vector Biolabs (Malvern, PA, USA). The pri-miR-204 sequence is located in the intron of the EF1a promoter that drives GFP expression, which will be used as a marker for detection of the *miR-204* expression introduced by AAV. We performed the surgery of partial meniscectomy on the right knee of wild type (WT) C57BL/6 mice to induce OA 10 days before they turned 3-month-old^33^. Ten days later, we performed intraarticular injection. Specifically, a longitudinal skin incision was made to visualize the patellar ligament and the patella; then we injected 5 × 10^9^ AAV particles in a 10 µl volume into the knee joint cavity by inserting a small needle into the area underneath the patella of the right leg.

### Tissue/cell isolation and culture

Mouse synovium were isolated from mouse knee joints^51^. Briefly, a skin incision was made to expose knee joints and quadriceps were resected and reversed distally to remove patella and patellar ligament. As a result, the synovium below patella were exposed for visualization and isolation. For synovial cell isolation, isolated synovium were incubated in 0.1% collagenase D (Roche, Indianapolis, IN, USA) for 15 min. Mouse articular chondrocytes were isolated according to a published protocol^52^ and cultured before analysis. Mouse bone marrow cells were isolated from long bones and cultured for 5 days in α-minimal essential medium (α-MEM) (Life Technologies, Grand Island, NY, USA) with 20% fetal bovine serum (FBS) (Life Technologies, Grand Island, NY, USA) and then in α-MEM with 10% FBS for a longer duration. CD45^−^ BMSC purification was performed by anti-CD45-mediated negative selection^22^. Specifically, fewer than 10^7^ cells were resuspended in 100 µl supplied buffer and incubated with 10 µl CD45 MicroBeads (Miltenyi Biotec, Cologne, Germany) for 15 min at 4–8 °C. After washing, the cell-MicroBead complexes were subjected to magnetic separation and the flow-through was resuspended in α-MEM with 10% FBS and plated in culture dishes. To induce osteoblast differentiation, after reaching 100% confluency, cells were cultured in α-MEM supplemented with 10% FBS, 10 nM dexamethasone, 50 µg/ml ascorbic acid and 10 mM β-glycerophosphate. Alkaline phosphatase (ALP) and Alizarin red staining were performed using 1-Step™ NBT/BCIP Substrate Solution (Thermo Fisher, Catalog number: 34042) and 0.5% Alizarin red Staining solution^11^. Von Kossa staining was performed by placing cell culture plates submerged with 1% silver nitrate under the UV lamp for 10 min.

### In vitro assays

For transfection of miRNA or siRNA oligos, Lipofectamine RNAi MAX transfection reagent (Life Technologies, Grand Island, NY, USA) was used and siRNAs against *Runx2*, or *Ngf* (Sigma-Aldrich, St. Louis, MO, USA) or *miR-204 mimic* (Dharmacon, Lafayette, CO, USA) were transfected at a concentration of 50 nM. Retroviral vectors for mouse *Ngf* overexpression were constructed using the forward primer for *Ngf-A* (5′-TATAGAATTCATGCTGTGCCTCAAGCCAGTG-3′) or for *Ngf-B* (5′-TATAGAATTCATGTCCATGTTGTTCTACACTC-3′) and the reverse primer (5′-TATACTCGAGTCAGCCTCTTCTTGTAGCCTT-3′, for both *Ngf* isoforms) to amplify two *Ngf* isoforms which were digested with *EcoR*I and *Xho*I and then ligated to pMX vector. The preparation and infection of retrovirus were performed as follows^53^. Briefly, Plat-E packaging cells were cultured in DMEM (Life Technologies, Grand Island, NY, USA) with 10% FBS and transfected with pMX retroviral vectors as described^11^. After 48 h, the retroviral supernatant was collected and used to infect cells. Selection was performed using 5 µg/ml puromycin (EMD Millipore, Billerica, MA, USA) for 2–3 weeks. Western blot analysis was performed^11^, using antibodies against Runx2 (1:1,000, D130-3, MBL, Woburn, MA, USA), β-actin (1: 10,000, A5441, Sigma-Aldrich, St. Louis, MO, USA), phospho-Akt Thr308 (1: 1000, #9275), total Akt (1: 3000, #9272), phospho-PI3 Kinase p85 (Tyr458)/p55 (Tyr199) (1:1000, #4228), and NGF (1:1000, #2046) (the last four antibodies all from Cell Signaling, Danvers, MA, USA). Uncropped and unprocessed scans of the most important blots are supplied in the Source Data file. CLIP assays were performed as follows^54^. Briefly, CD45^−^ BMSCs were rinsed with PBS, then irradiated at 400 mJ cm^−2^ (setting of 4000 and *λ* = 254 nm). Then we lysate the cells on ice for 10 min in 1 ml buffer with 50 mM Tris-HCl (pH 7.8), 300 mM NaCl, 1% NP-40, 5 mM EDTA (pH 8.0) and 10% glycerol, 5 mM β-mercaptoethanol and protease inhibitors. Cleared lysates will be incubated with anti-Ago (mouse anti-Ago 2A8, Millipore, cat. no. MABE56) magnetic beads for 1 h and then washed with lysis buffer for three times. The Ago-binding beads were then treated with diluted RNase, T4 PNK, T4 RNA ligase and Shrimp Alkaline Phosphatase sequentially. Qiagen miRNeasy kit was used to purify RNA from the beads and reverse transcription was performed using qScript microRNA cDNA Synthesis Kit (Quantabio). PCR were then performed using primer pairs covering miR-204 and putative binding sites of Runx2 3′-UTR. For profiling gene expressions, quantitative RT-PCR was performed, using the primer pairs for *Runx2* (5′-CAAGAAGGCTCTGGCGTTTA-3′ and 5′-TGCAGCCTTAAATGACTCGG-3′), *Alp* (5′-CCAACTCTTTTGTGCCAGAGA-3′ and 5′-GGCTACATTGGTGTTGAGCTTTT-3′), *osterix* (5′-AGAGGTTCACTCGCTCTGACGA-3′ and 5′-TTGCTCAAGTGGTCGCTTCTG-3′), *osteocalcin* (5′-CTGACCTCACAGATGCCAAGC-3′ and 5′-TGGTCTGATAGCTCGTCACA AG-3′), *p16* (5′-GAACTCTTTCGGTCGTACCC-3′ and 5′-GTTCGAATCTGCACCGTAGT-3′), *p21* (5′-CCTGGTGATGTCCGACCTG-3′ and 5′-CCATGAGCGCATCGCAATC-3′), *p27* (5′-TCAAACGTGAGAGTGTCTAACG-3′ and 5′-CCGGGCCGAAGAGATTTCTG-3′), *p57* (5′-CGAGGAGCAGGACGAGAATC-3′ and 5′-GAAGAAGTCGTTCGCATTGGC-3′), *Mmp13* (5′-CTTCTTCTTGTTGAGCTGGACTC-3′ and 5′-CTGTGGAGGTCACTGTA GACT-3′), *Adamts5* (5′-GGAGCGAGGCCATTTACAAC-3′ and 5′-CGTAGACAAGGTAG CCCACTTT-3′), *Ngf* (5′-ACTGGACTAAACTTCAGCAT TCC-3′ and 5′-GGGCAGCTATT GGTGCAGTA-3′) and *β-actin* (5′-GGCTGTATTCCCCTCCATCG-3′ and 5′-CCAGTTGG TAACAATGCCATGT-3′). Specifically, for mouse *miR-204* and *miR-211* qRT-PCR, the reverse transcription primer is *mmu*-*miR-204*/*-211-RT* (5′-GTCGTATCCAGTGCAGGGTCCGAGG TATTCGCACTGGATACGACAGGCAW-3′), and the PCR primers are *mmu*-*miR-204* forward (5′-GGGCTTCCCTTTGTCATCCTAT-3′), *mmu-miR-211* forward (5′- GGGCTTCCCTTT GTCATCCTT-3′) and the reverse primer (5′-CCAGTGCAGGGTCCGAGGT-3′).

### Micro-CT, histology, ISH, IHC and histomorphometry

We used a Scanco µCT35 scanner (Scanco Medical, Brüttisellen, Switzerland) with 55 kVp source and 145 μAmp current for formalin-fixed mouse legs with a resolution of 10 μm. The scanned images from each group were evaluated at the same thresholds to allow three-dimensional structural rendering of each sample. For histology, ISH and IHC, tissues were fixed in 10% formalin, decalcified, and embedded in paraffin. Serial sagittal sections of knee joints were cut every 3 μm from the medial compartments. The sections were stained with Alcian blue/hematoxylin & orange G (AB/H&OG) for histological analysis. OARSI scoring was performed to evaluate knee joint AC destruction essentially^55^. Specifically, both medial femoral condyle and medial tibial plateau were analyzed through three-level sections of the joints and the severity of OA is expressed as the summed scores for the entire joint.

We performed in situ hybridizations on 3-μm-thick sections using the microRNA ISH Buffer and Controls Kit (Exiqon, Vedbaek, Denmark). Briefly, sections were deparaffinized, permeabilized with Proteinase K for 10 min at 37 °C, treated with 3% H_2_O_2_ to block endogenous peroxidase activity and then dehydrated before applying 40 nM double-DIG LNA™ microRNA probe for *miR-204* or *miR-211*, both of which were purchased from Exiqon. Hybridization was performed for 1 h at 53 °C for *mmu-miR-204*, and 48 °C for *mmu-miR-211* respectively. After serial washes with 5× SSC buffer, 1× SSC buffer and 0.2× SSC buffer, sections were incubated with blocking solution for 15 min, and then applied with anti-DIG-POD antibodies (Roche Applied Science, Indianapolis, IN, USA). Lastly, TSA-plus FITC substrate (Perkin Elmer, Waltham, MA, USA) was applied for detection of fluorescent signal and VECTASHIELD Mounting Medium with DAPI (Vector Laboratories, Burlingame, CA, USA) was used to mount the slides. For IHC, 3 μm paraffin sections were heated at 95 °C in Antigen Unmasking Solution (Vector Laboratories, Burlingame, CA, USA) for 10–15 min, and then sequentially treated with 3% H_2_O_2_, 0.5% Triton X-100, Avidin/Biotin Blocking Kit (Invitrogen, Carlsbad, CA, USA). After blocking with 10% normal goat serum (Vector Laboratories, Burlingame, CA, USA) for 1 h, sections were treated with 1/200 anti-Runx2 antibody (D130-3, MBL, Woburn, MA, USA), 1/200 phospho-Akt antibody (#9275, Cell Signaling, Danvers, MA, USA), 1/2000 PCNA antibody (ab29, Abcam, Cambridge, MA, USA), 1/200 MMP13 antibody (MAB13424, EMD Millipore, Billerica, MA, USA), 1/500 Adamts5 antibody (ab41037, Abcam, Cambridge, MA, USA), 1/1000 Cre antibody (69050, Novagen, Billerica, MA, USA), 1/200 NGF antibody (ab6199, Abcam, Cambridge, MA, USA), or 1/500 Adamts4 (ab185722, Abcam, Cambridge, MA, USA) antibody overnight at 4 °C and incubated with 1/400 secondary biotinylated goat anti-rabbit or anti-mouse antibody (Vector Laboratories, Burlingame, CA, USA) for 30 min, followed by treatment with VECTASTAIN Elite ABC Kit (Vector Laboratories, Burlingame, CA, USA). IHC signals were revealed by ImmPACT DAB Peroxidase Substrate (Vector Laboratories, Burlingame, CA, USA). For fluorescence immunostaining, sections were incubated with 1/250 Ki67 antibody (ab15580, Abcam, Cambridge, MA, USA) overnight at 4 °C and then incubated with secondary antibody conjugated to Alexa Fluor 488 (Invitrogen, Carlsbad, CA, USA) for 30 min. Images of histology, ISH and IHC were captured using CellSens Imaging Software (Olympus) on an Olympus BX43 microscope, or a Zeiss LSM700 confocal microscope. Histomorphometric measurements were performed using OsteoMeasure software (OsteoMetrics, Inc., Atlanta, GA, USA).

### Flow cytometry

After 2-week culture in α-MEM plus 10% FBS, mouse BMSCs were trypsinized, pelleted and then resuspended in PBS containing 2% FBS to a concentration of 2 × 10^6^ cells/ml. Freshly isolated bone marrow cells were resuspended to a concentration no more than 1 × 10^7^ cells/ml. Cells were stained with anti-mouse CD45 PerCP-Cy5.5 (eBioscience, 45-0451-80), anti-mouse/rat CD29 PE-Cy7 (eBioscience, 25-0291-80), anti-mouse CD105 (Endoglin) PE (eBioscience, 12-1051-81), and anti-mouse Ly-6A/E (Sca-1) APC (eBioscience, 17-5981-81), and analyzed with an LSRII cytometer (Becton Dickinson, San Jose, CA, USA). For BrdU Incorporation, the mice were intraperitoneally labeled twice (a 16-h interval) with 1 mg/mouse/injection BrdU and euthanized 2 h after the second injection. Freshly harvested bone marrow was treated with 1× RBC Lysis Buffer (eBiosciences, San Diego, CA, USA). The remaining cells were stained with anti-mouse CD45 PerCP-Cy5.5, anti-mouse/rat CD29 PE-Cy7, anti-mouse CD105 (Endoglin) PE, and anti-mouse Ly-6A/E (Sca-1) Alexa Fluor 700 (eBioscience, 56-5981-82) before permeabilization, DNase treatment and staining with anti-BrdU APC according to the instruction of APC BrdU Flow Kits (BD Pharmagen, Franklin Lakes, NJ, USA). The percentage of BrdU-positive fractions was assessed using FlowJo analysis software (Tree Star, Ashland, OR, USA).

### Animal behavioral tests

Testing for mechanical allodynia (von Frey sensitivity) was performed using a calibrated set of von Frey filaments (Stoelting, Wood Dale, IL)^56^. Before the von Frey test, we allowed animals to adapt to the environment, including an elevated mesh platform for 15 min. The set of von Frey filaments was used to poke from below to the hind paw to calculate the 50% force withdrawal threshold using an iterative approach. The tests were performed in a blind manner that the investigator is not aware of the identification of animals as well as the study groups.

### Experimental design and statistical analyses

The sample size for each experiment was determined based on our previous experiences. All the data were expressed as mean ± s.d., as indicated in the figure legends. Statistical analyses were completed with Prism GraphPad. Unpaired Student’s *t* test (for two groups), one-way ANOVA (for multiple groups) and two-way ANOVA (for multiple groups and time points) were used followed by the Tukey−Kramer test. *P* *<* 0.05 was considered statistically significant.

## Supplementary information

Supplementary Information

Supplementary Data 1

## Data Availability

The data that support the findings of this study are available within the article and its Supplementary Information files or from the corresponding author upon reasonable request.
