# Supplementary material for: The microRNAs miR-204 and miR-211 maintain joint homeostasis and protect against osteoarthritis progression
Source: Nat Commun. 2019 Jun 28;10:2876. doi: 10.1038/s41467-019-10753-5 (PMC6599052; doi:10.1038/s41467-019-10753-5)
Supplement: Supplementary file 1 — Supplementary Information [file 41467_2019_10753_MOESM1_ESM.pdf]

Supplementary Information

The microRNAs *miR-204* and *miR-211* maintain joint homeostasis and protect against osteoarthritis progression

Jian Huang[1], Lan Zhao[1], Yunshan Fan[1], Lifan Liao[1], Peter X. Ma[2], Guozhi Xiao[1], Di Chen[1]

Supplementary Table 1. Significant Histologic findings in non-limb organs of three female *miR-204*$^{-/-}$*; miR-211*$^{-/-}$ mice by the Mouse Metabolic Phenotyping Center.

Age	15 months	10 months	7.5 months
Spleen	White pulp disorganization with fusion of follicles, absent mantle zone and possible hyperplastic marginal zone	Extramedullary hematopoiesis; Marked marginal zone hyperplasia	Mild white pulp disorganization with possible absent mantle zone
Kidneys	Minimal chronic lymphoplasmacytic pyelitis	Minimal chronic lymphoplasmacytic pyelitis	Mild intrapelvic mineral
Heart	Valvular endocardiosis	Valvular endocardiosis	Valvular endocardiosis
Skin / Connective tissue	Moderate follicular dysplasia with regional follicular atrophy; Atrophy of fat	Moderate follicular dysplasia with regional follicular atrophy	Moderate follicular dysplasia with regional follicular atrophy
Eyes	Hypermature cataracts with rupture, retinal dysplasia and uveal melanosis	Hypermature cataracts with rupture, posterior synechiae, retinal dysplasia, detachment and uveal melanosis	No significant histologic changes were observed

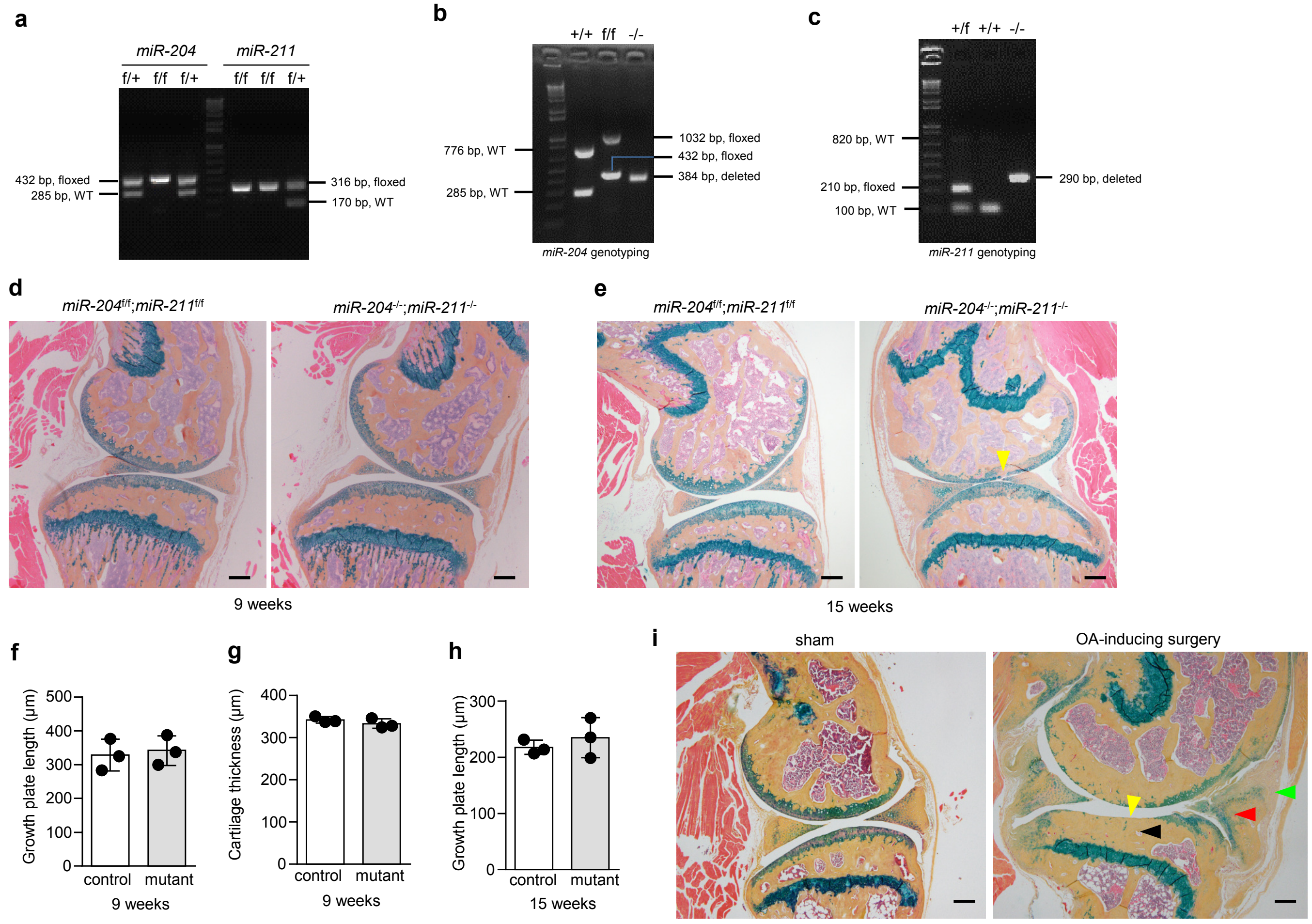

Supplementary Fig. 1 Genotyping and phenotyping of *miR-204* and *miR-211* floxed and deleted alleles. **a** PCR analysis was performed to confirm genotypes of floxed *miR-204* (left) and *miR-211* alleles (right). The PCR products for different alleles are labeled and their sizes are denoted. **b** PCR analysis of the deleted alleles of *miR-204*. The PCR products for different alleles are labeled and their sizes are denoted. **c** PCR analysis of the deleted alleles of *miR-211*. The PCR products for different alleles are labeled and their sizes are denoted. Note that the floxed *miR-211* allele can also generate a 1076-bp band by theory, which is difficult to be produced by regular PCR due to its rich GC content. **d, e** Representative histology images of knee joints of 9-week-old (**d**) and 15-week-old (**e**) *miR-204*/*-211* floxed mice and global double KO (*miR-204*$^{-/-}$; *miR-211*$^{-/-}$) mice stained by Alcian blue/Orange G. Yellow arrowhead, loss of articular cartilage. n = 5. Scale bar, 200 μm. **f**, **h** Measurement of tibial growth plate length in 9-week-old (**f**) and 15-week-old (**h**) *miR-204*/*-211* floxed mice (control) and *miR-204*$^{-/-}$; *miR-211*$^{-/-}$ mice (mutant). n = 3. Unpaired Student's *t*-test. Error bars, s.d. **g** Measurement of articular cartilage thickness at the highest point in the tibial plateau of 9-week-old control and mutant mice. n = 3. Unpaired Student's *t*-test. Error bars, s.d. **i** Representative histology images of knee joints of the mice that received the OA-inducing surgery 6 months ago, to show histologic features of the OA model. Yellow arrowheads, loss of articular cartilage; red arrowheads, osteochondrophytes; green arrowheads, synovial hyperplasia; black arrowheads, subchondral sclerosis. n = 5. Scale bar, 200 μm.

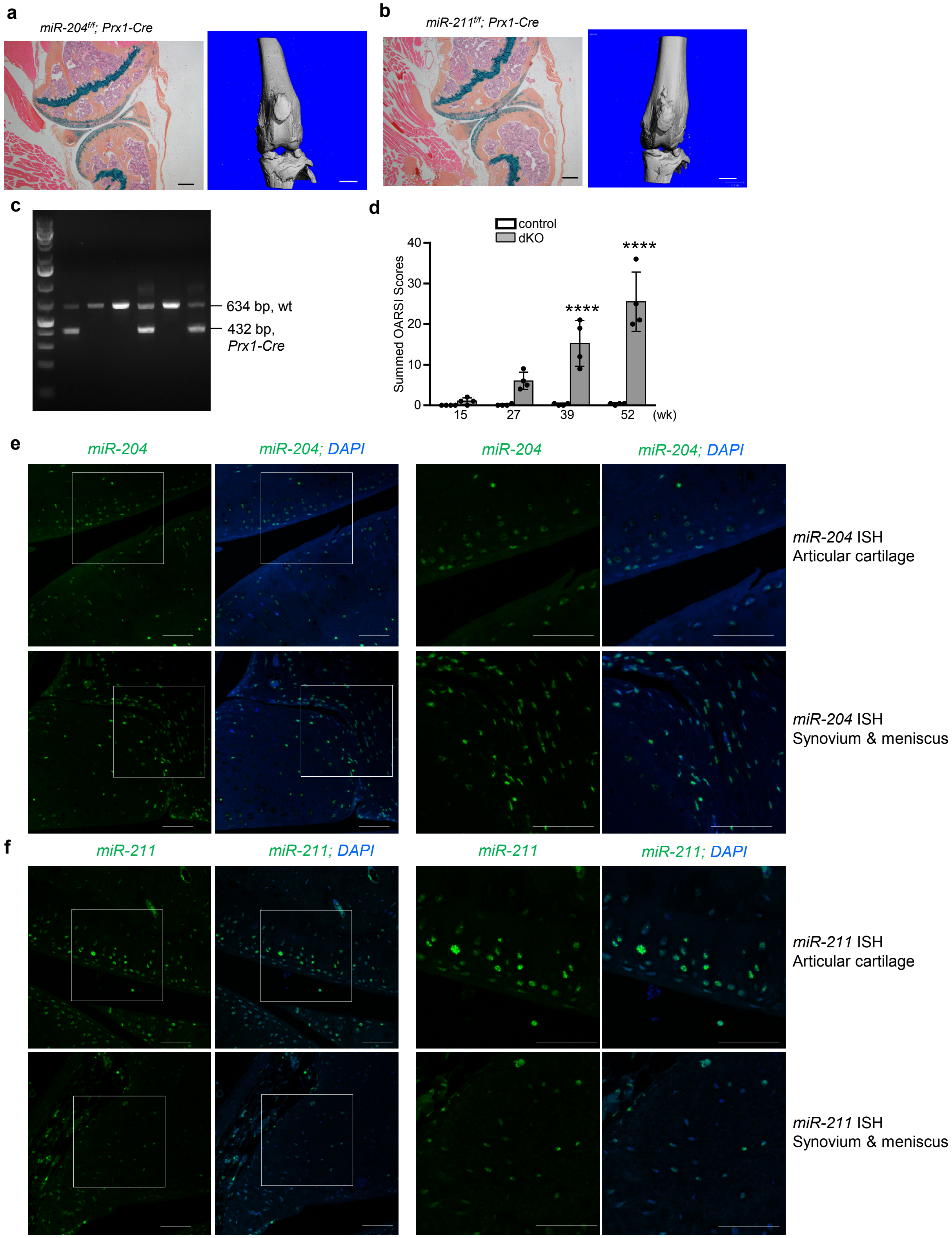

Supplementary Fig. 2

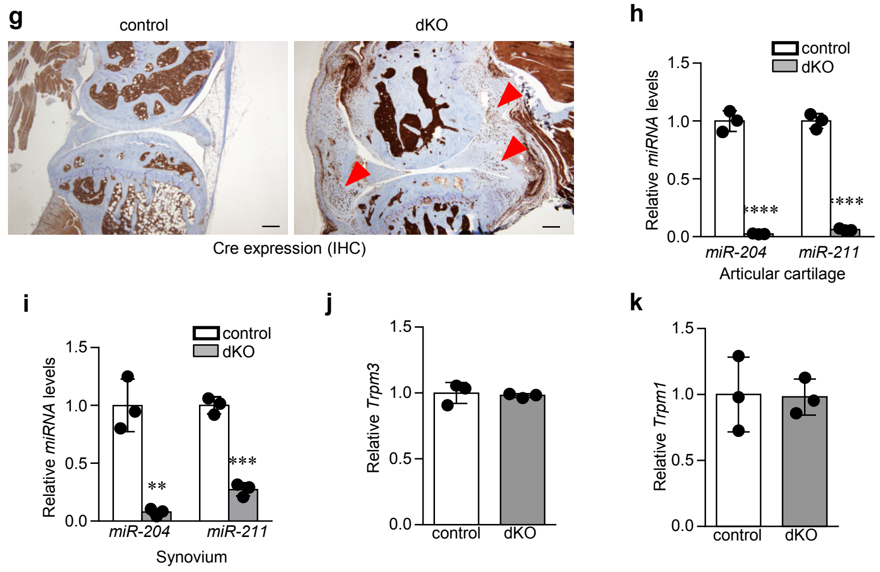

Supplementary Fig. 2. Deficiency of *miR-204*/*-211* in mesenchymal stem/progenitor cells recapitulates OA pathogenesis. **a**, **b** Representative histology (left) and μCT (right) images of knee joints of 42-week-old single *miRNA* conditional knockout mice (*miR-204*$^{f/f}$; *Prx1-Cre or miR-211*$^{f/f}$; *Prx1-Cre*). Scale bar in histology images, 300 μm. Scale bar in μCT images, 1 mm. **c** PCR analysis of the *Prx1-Cre* transgene-inserted locus on Chromosome 7. The PCR products for different alleles are labeled and their sizes are denoted. Three primers used in the PCR reaction are ACACCTCCCCCTGAACCTGA, GACACTCCCCACCCCTTCTA, and TGTTTGCATTTGTTGCTTTT, generating 402 bp for *Prx1-Cre* and 634 bp for wide type DNA. **d** Longitudinal analysis of knee joint of *miR-204*/*-211* dKO mice by histology. Analysis using Osteoarthritis Research Society International (OARSI) scoring system was performed to evaluate knee joint articular cartilage (AC) destruction in different ages of control and dKO mice. Both medial femoral condyle and medial tibial plateau were analyzed through three-level sections of the joints and the severity of OA is expressed as the summed scores for the entire joint. The results of these data were analyzed by two-way ANOVA. n = 4. **e** ,**f** Representative confocal microscopy images of in situ hybridization using LNA-enhanced *miR-204* (**e**) or *miR-211* (**f**) probe (green) on knee joint sections of 7-month-old WT mice. The right panels are the enlargements of the images on the left side as indicated by the box. DAPI staining marks nuclei (blue). n = 5. Scale bar, 50 μm. **g** IHC analysis of Cre expression in *miR-204*/*-211* dKO mice. IHC results demonstrate Cre expression in the synovium, AC, and meniscus of knee joints of dKO mice (right panel). *Prx1-Cre* negative mice were used as the control. Red arrowheads, Cre-positive cells. n = 5. Scale bar, 200 μm. **h, i** Quantitative RT-PCR analysis of *miR-204* and *miR-211* expression in AC (**h**) and synovium (**i**) of joint tissues from control or dKO mice. Unpaired Student's *t*-test. n = 3. Error bars, s.d. **j, k** Quantitative RT-PCR analysis of *Trpm3* and *Trpm1* expression in AC from control (Ctrl) or dKO mice. n = 3. Error bars, s.d. * $P < 0.05$, ** $P < 0.01$, *** $P < 0.001$, **** $P < 0.0001$.

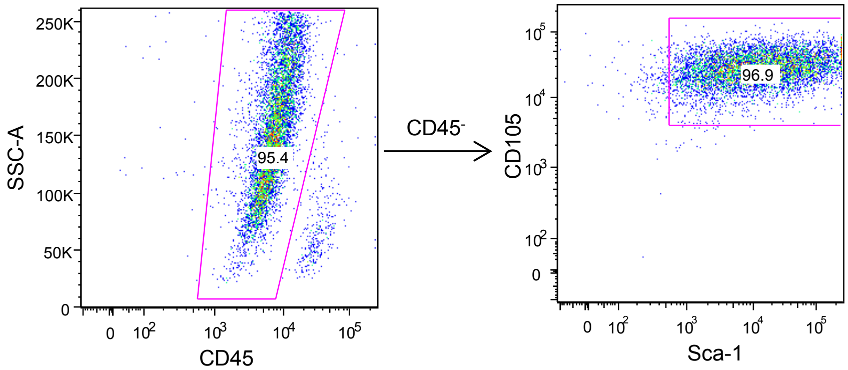

Supplementary Fig. 3 Identification of $CD45^-$ BMSCs as MPCs. Representative flow cytometry profile of CD45, CD105 and Sca-1 expression in purified WT $CD45^-$ BMSCs. The data show that 95.4% of purified $CD45^-$ BMSCs do not express CD45 and most of them express markers like CD105 and Sca-1. n = 3.

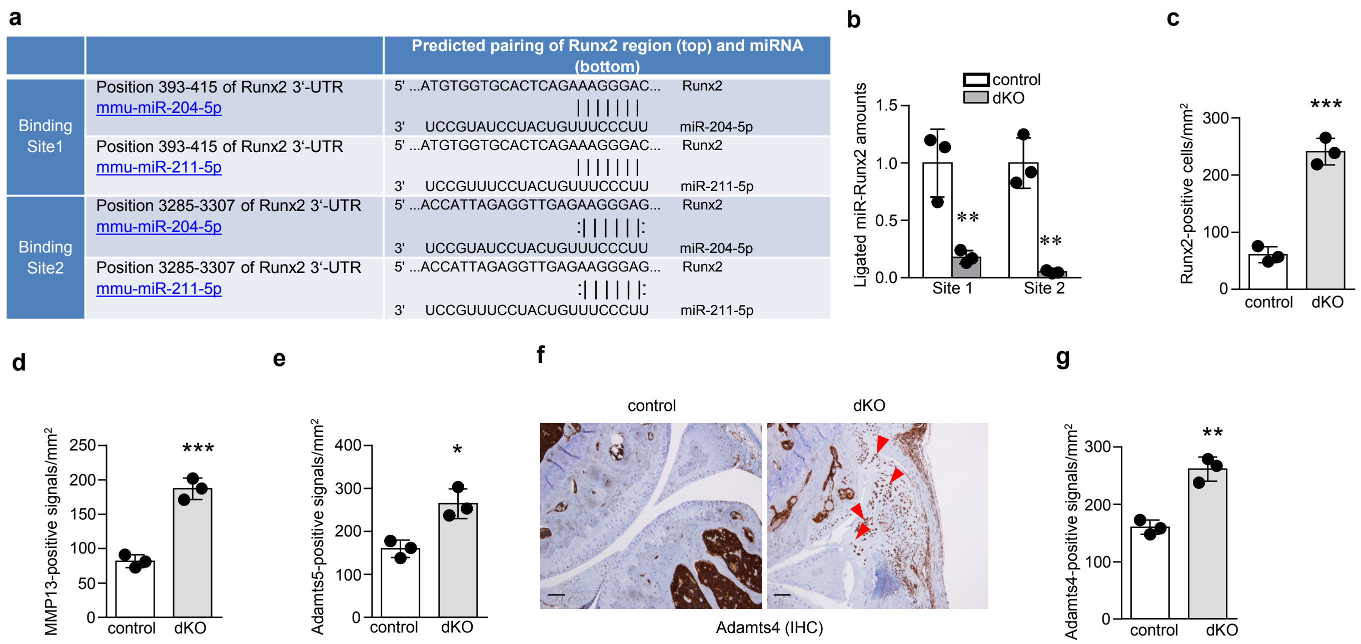

Supplementary Fig. 4. *miR-204* and *miR-211* bind to *Runx2* mRNA and their deficiency upregulates Runx2 and cartilage-catabolic enzymes. **a** Two *miR-204*/*-211*-binding sites were identified in the 3'-UTR of *Runx2* mRNA. **b** qRT-PCR was performed to measure *miR-204*/*-211-Runx2* chimeras in control or dKO cells after cross-linking and immunoprecipitation (CLIP) assays. ** $P < 0.01$, unpaired Student's *t*-test. n = 3. Error bars, s.d. **c, d, e, g** Quantification of positive immunohistochemistry signals per mm^2 of knee joints for detection of Runx2 (**c**), MMP13 (**d**), Adamts5 (**e**), and Adamts4 (**g**). n = 3. * $P < 0.05$, ** $P < 0.01$, *** $P < 0.001$, unpaired Student's *t*-test. **f** IHC results of Adamts4 in control and dKO mice. Red arrowheads, Adamts4-positive cells. Scale bar, 100 μm.

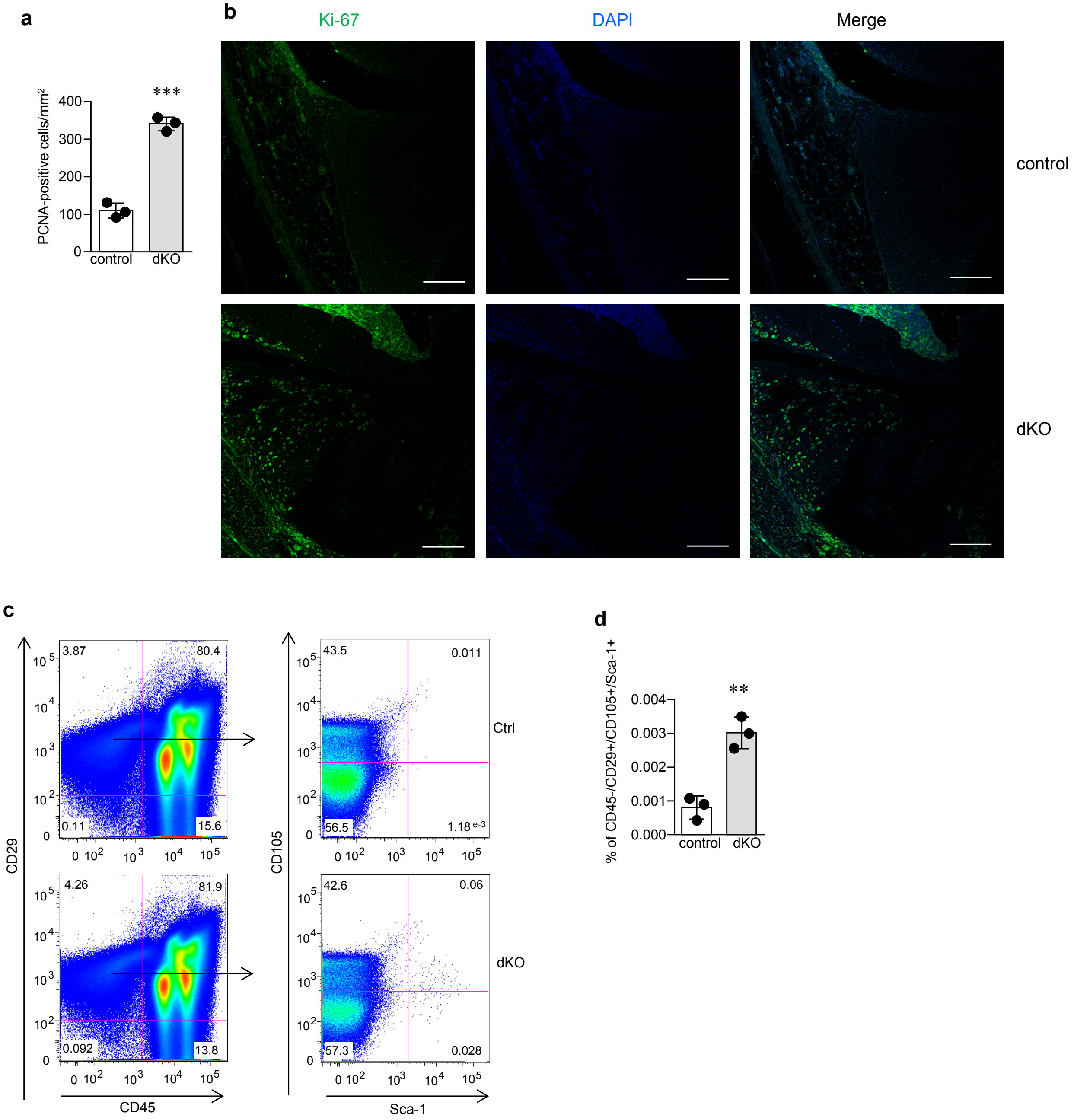

Supplementary Fig. 5 Proliferation of mesenchymal stem/progenitor cells are enhanced in *miR-204/-211* dKO mice. **a** Quantification of cells with positive immunohistochemistry signals per mm^2 of knee joints for detection of PCNA. n = 3. *** P < 0.001, unpaired Student's t-test. **b** Representative confocal microscopy images of immunostaining of Ki-67 (green) on knee joint sections of control and dKO mice. n = 5. Scale bar, 50 μm. **c**, **d** Representative flow cytometry profile (**c**) and quantification (**d**) of CD45, CD29, CD105 and Sca-1 expression in bone marrow cells freshly isolated from Control and dKO mice. ** P < 0.01, unpaired Student's t-test. n = 3.

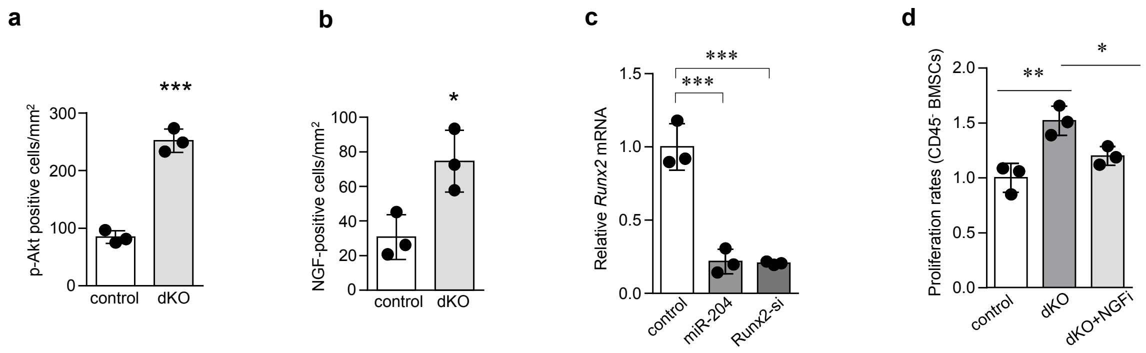

Supplementary Fig. 6 The *miR-204*/*-211*-Runx2 axis in regulation of nerve growth factor (NGF)-Akt signaling and mesenchymal progenitor cell proliferation. **a**, **b** Quantification of cells with positive immunohistochemistry signals per mm^2 of knee joints for detection of p-Akt (**a**) and NGF (**b**). n = 3. * $P < 0.05$, *** $P < 0.001$, unpaired Student's *t*-test. **c** Quantitative RT-PCR analysis of *Runx2* expression in mouse $CD45^-$ BMSCs transfected with *miR-204* or *Runx2* siRNA. *** $P < 0.001$, one-way ANOVA followed by the Tukey-Kramer test. n = 3. Error bars, s.d. **d** *NGF* knockdown reduced cell proliferation in dKO $CD45^-$ BMSCs determined by cell number counts. * $P < 0.05$, ** $P < 0.01$, one-way ANOVA followed by the Tukey-Kramer test. n = 3.

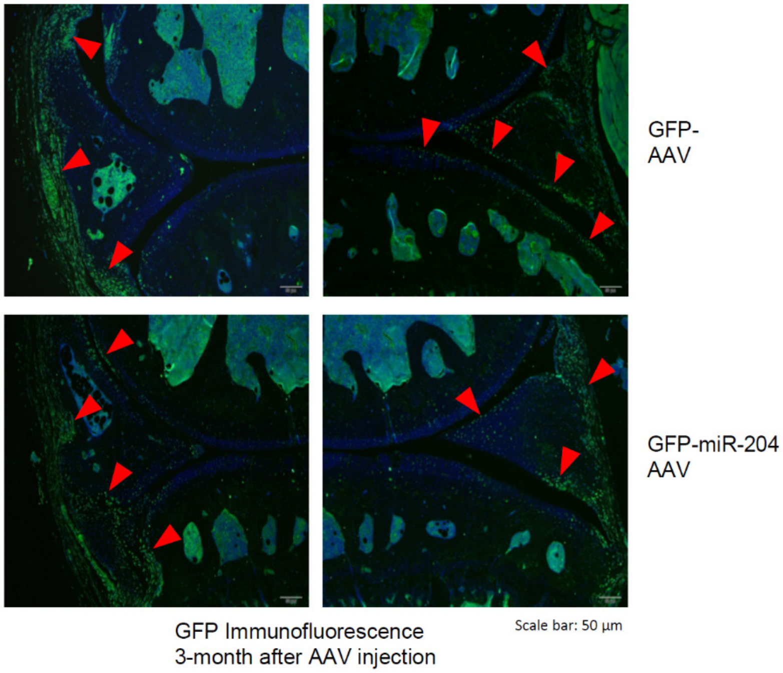

Supplementary Fig. 7 Immunofluorescence (IF) of GFP expression in joint tissue after AAV5-miR-204 administration. IF data showed GFP expression in the synovium, articular cartilage, and meniscus of knee joints of 6-month-old C57BL/6 mice that received AAV5-GFP or AAV5-miR-204 injections at the age of 3 months. Both AAV5-GFP and AAV5-miR-204 express GFP as a reporter of AAV5 infection efficiency. n = 5. Red arrowheads, GFP-positive cells.

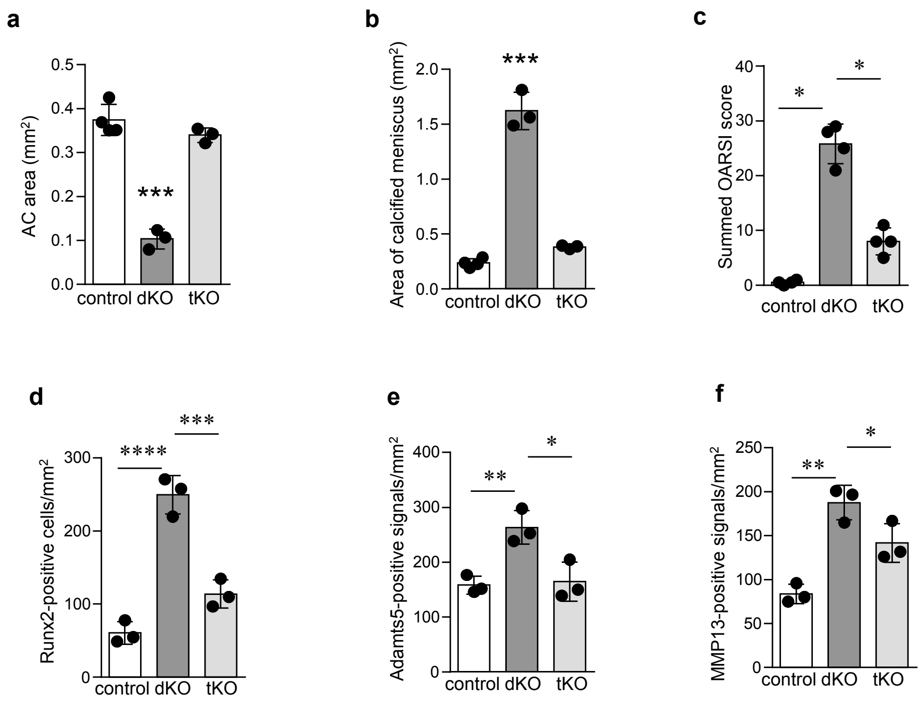

Supplementary Fig. 8 Heterozygous deletion of *Runx2* mitigates the OA phenotype of the dKO Mice. **a** Measurement of articular cartilage area (mm^2) of control, dKO and triple KO (tKO) (*miR-204*$^{flox/flox}$; *miR-211*$^{flox/flox}$; *Runx2*$^{+/f}$; *Prx1-Cre*) knee joints. n = 3 or 4. **b** Histomorphometric quantification of calcified meniscus area (mm^2) of knee joints from control, dKO and tKO mice, which are used to denote osteophyte formation around meniscus. n = 3 or 4. **c** Analysis using OARSI scoring system was performed to evaluate knee joint AC destruction in the dKO or tKO mice as indicated. Mann-Whitney test between two groups. n = 4. **d**-**f** IHC results of Runx2, Adamts5, and MMP13 expression in the dKO or tKO mice . n = 3. * $P < 0.05$, ** $P < 0.01$, *** $P < 0.001$, **** $P < 0.0001$, one-way ANOVA followed by the Tukey-Kramer test. Error bars, s.d.
