# Supplementary figures and images for: The microRNAs miR-204 and miR-211 maintain joint homeostasis and protect against osteoarthritis progression
Source: Nat Commun. 2019 Jun 28;10:2876. doi: 10.1038/s41467-019-10753-5 (PMC6599052; doi:10.1038/s41467-019-10753-5)

Full unedited gel for Figure 3b

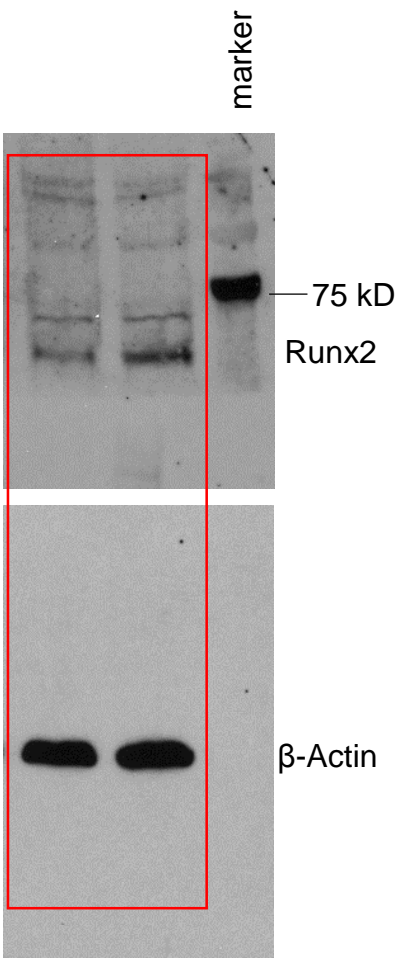

Full unedited gel for Figure 5

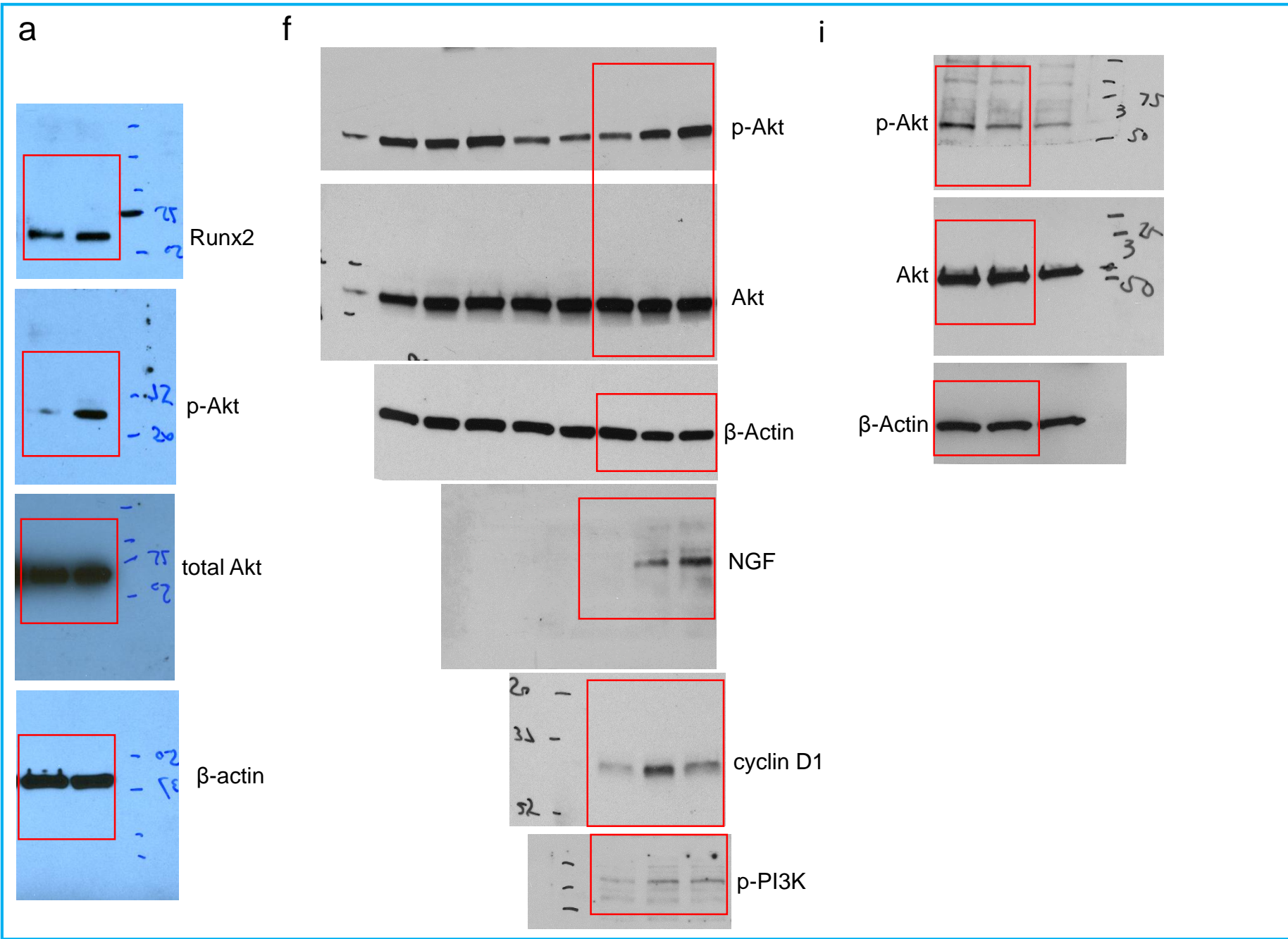

Supplement: Supplementary file 2 — Supplementary Data 1 [file 41467_2019_10753_MOESM2_ESM.pdf]
